# Prehabilitative resistance exercise reduces neuroinflammation and improves mitochondrial health in aged mice with perioperative neurocognitive disorders

**DOI:** 10.1186/s12974-022-02483-1

**Published:** 2022-06-15

**Authors:** Yan Liu, John Man Tak Chu, You Ran, Yan Zhang, Raymond Chuen Chung Chang, Gordon Tin Chun Wong

**Affiliations:** 1 Department of Anaesthesiology, LKS Faculty of Medicine, The University of Hong Kong, Pokfulam, Hong Kong, SAR China; 2 Laboratory of Neurodegenerative Diseases, School of Biomedical Sciences, LKS Faculty of Medicine, The University of Hong Kong, L4-49, Laboratory Block, 21 Sassoon Road, Hong Kong, SAR China; 3 State Key Laboratory of Brain and Cognitive Sciences, The University of Hong Kong, Hong Kong, SAR China; 4 Department of Rehabilitation Medicine, The Third Affiliated Hospital of Sun Yat-Sen University, Guangzhou, China; 5 Department of Anaesthesiology, The University of Hong Kong, K424, Queen Mary Hospital, Hong Kong, SAR China

**Keywords:** Resistance exercise, Neuroinflammation, Perioperative neurocognitive disorders, Mitochondrial function, Synaptic deficit

## Abstract

**Background:**

Postoperative neurocognitive dysfunction remains a significant problem in vulnerable groups such as the elderly. While experimental data regarding its possible pathogenic mechanisms accumulate, therapeutic options for this disorder are limited. In this study, we evaluated the neuroprotective effect of a period of preconditioning resistant training on aged mice undergoing abdominal surgery. Further, we examined the underlying mechanisms from the perspective of neuroinflammatory state and synaptic plasticity in the hippocampus.

**Methods:**

18-month-old C57BL/6N mice were trained for 5 weeks using a ladder-climbing protocol with progressively increasing weight loading. Preoperative baseline body parameters, cognitive performance and neuroinflammatory states were assessed and compared between sedentary and trained groups of 9-month-old and 18-month-old mice. To access the neuroprotective effect of resistance training on postoperative aged mice, both sedentary and trained mice were subjected to a laparotomy under 3% sevoflurane anesthesia. Cognitive performance on postoperative day 14, hippocampal neuroinflammation, mitochondrial dysfunction and synaptic plasticity were examined and compared during groups.

**Results:**

18-month-old mice have increased body weight, higher peripheral and central inflammatory status, reduction in muscle strength and cognitive performance compared with middle-aged 9-month-old mice, which were improved by resistance exercise. In the laparotomy group, prehabilitative resistant exercise improved cognitive performance and synaptic plasticity, reduced inflammatory factors and glial cells activation after surgery. Furthermore, resistance exercise activated hippocampal PGC-1α/BDNF/Akt/GSK-3β signaling and improved mitochondrial biogenesis, as well as ameliorated mitochondrial dynamics in postoperative-aged mice.

**Conclusions:**

Resistance exercise reduced risk factors for perioperative neurocognitive disorders such as increased body weight, elevated inflammatory markers, and pre-existing cognitive impairment. Accordantly, preoperative resistance exercise improved surgery-induced adverse effects including cognitive impairment, synaptic deficit and neuroinflammation, possibly by facilitate mitochondrial health through the PGC1-a/BDNF pathway.

**Supplementary Information:**

The online version contains supplementary material available at 10.1186/s12974-022-02483-1.

## Introduction

Adverse neurocognitive sequelae following surgery in older patient has been recognized as early as the 1950s [1]. The recently coined term perioperative neurocognitive disorders (PNDs) include cognitive decline diagnosed before or up to 12 months after surgery [2]. PNDs affect multiple cognitive domains [3], and are associated with an increased length of hospitalization [4] and long-term dementia [5]. With a global aging population and increasing number of operations performed in the elderly [6], effective options to mitigate PNDs are urgently required.

Neuroinflammation is closely associated with PNDs as well as other neurodegenerative disease [7]. The hippocampus region is important to cognition and is particularly vulnerable to age-related insults [8]. Chronic neuroinflammation is seen in both aged animals and humans, and the severity of inflammation can be aggravated by surgical trauma, leading to synaptic dysfunction and apoptosis [9]. This biological sequence in the brain is regulated by activated microglia and astrocytes and glial activation can persist and accelerate cognitive decline in susceptible populations. From previous reports, activation of glial cells can lead to subsequent release of pro-inflammatory mediators, which in turn trigger mitochondrial and synaptic dysfunction in various neurodegenerative diseases [10]. In Alzheimer’s disease (AD), activated microglia releasing inflammatory cytokines and mitochondrial dysfunction are seen in the hippocampus [11]. In other reports, targeting the actions of specific pro-inflammatory cytokines, including IL-1 [12] and IL-6 [13], have been shown to attenuate postoperative cognitive impairment. Therefore, the presence of neuroinflammation correlates with both mitochondrial and synaptic dysregulation and reducing neuroinflammation may impede these changes and ameliorate cognitive dysfunction.

Exercise benefits age-related cognitive changes as well as early-stage dementia [14], and potentially has a powerful anti-inflammatory effect in neurodegeneration diseases. Specifically, exercise may reduce neuroinflammation, either by attenuating activation of microglia and astrocytes [15] or by reducing pro-inflammatory adipokines secretion in crosstalk between muscle and adipose tissue [16]. Physical exercise can regulate innate inflammatory responses via the hypothalamic pituitary adrenal axis and sympathetic nervous system [17]. Moreover, the beneficial effect of physical exercise on neurodegenerative diseases is associated with improving mitochondrial function and genesis [18].

Aerobic and resistance exercise are known to produce cardiovascular benefits and muscle hypertrophy, respectively [19]. With most studies on the neuroprotective effect of exercise focusing on the aerobic type, the effect of resistance exercise on cognition and synaptic function, particularly in PNDs, remained largely unknown. From a certain point of view, resistance exercise is efficacious and safe for preoperative patients. Resistance type exercises may be performed from a stationary position and theoretically reduces risks of fall in frailer patients. Furthermore, preoperative resistance exercise may increase muscle mass, thus minimizing perioperative muscle loss, that in turn might benefit postoperative rehabilitation. In light of these findings, we hypothesized that preoperative resistance exercise improves perioperative neurocognitive function and related neuropathological changes through modulating inflammatory responses and mitochondria dysfunction. An aged mice model was used as advanced age is a risk factor for PNDs and reduced cognitive reserves in the aged may render them more susceptible to the detrimental effects of neuroinflammation.

## Materials and methods

### Animal handling, assessment and killing

Male C57BL6/N mice aged 9 months or 18 months were housed in the Laboratory Animal Unit at the University of Hong Kong, which is fully accredited by the Association for Assessment and Accreditation of Laboratory Animal Care International. They were first acclimatized for a week to a 12/12 h light/dark cycle at temperatures between 20 and 22 °C and humidity of 50 ± 10%, with access to food and water ad libitum. The experimental protocols were approved by the Committee on the Use of Live Animals in Teaching and Research (CULATR) in The University of Hong Kong (CULATR No. 4192-16). In accordance with the 3Rs, all efforts were made to minimized the number and suffering of mice during experiments.

We first compared the general conditions, muscle strength and cognitive performances of 18-month-old mice with 9-month-old counterparts, dividing them into sedentary (9M-SED and 18M-SED) and resistance exercise (9M-RE, 18M-RE) groups. We then randomly divided the 18-month-old mice into control, laparotomy only (Lap), resistance exercise (RE), and resistance exercise followed by laparotomy (RE + Lap) groups to characterize the effect of resistance exercise on behavioral and pathological changes following surgery. In total, 122 aged mice were used to illustrate the effect of resistance exercise on PNDs, with 24 mice used to evaluated early regulation of inflammation and intracellular signal pathways, 66 mice used for behavioral tests, late regulation of inflammation, intracellular signal pathways and Golgi staining, 32 mice used to investigate changes in mitochondrial markers and morphology.

The body weight of the mice, the weight of the food and water provided for each cage on day 1 and day 3 of each week were recorded at the beginning and the end of the training period. Food and water intake for each mouse was calculated by averaging total consumption of each cage divided by the number of occupants.

After behavioral testing, the mice were killed by CO_2_ asphyxiation in accordance with the guidelines of the American Veterinary Medical Association. Blood samples were collected from the heart before it was perfused with saline. The brain, liver and gastrocnemius tissues were subsequently harvested and processed.

### Resistance training protocol

**Fig. 1:**
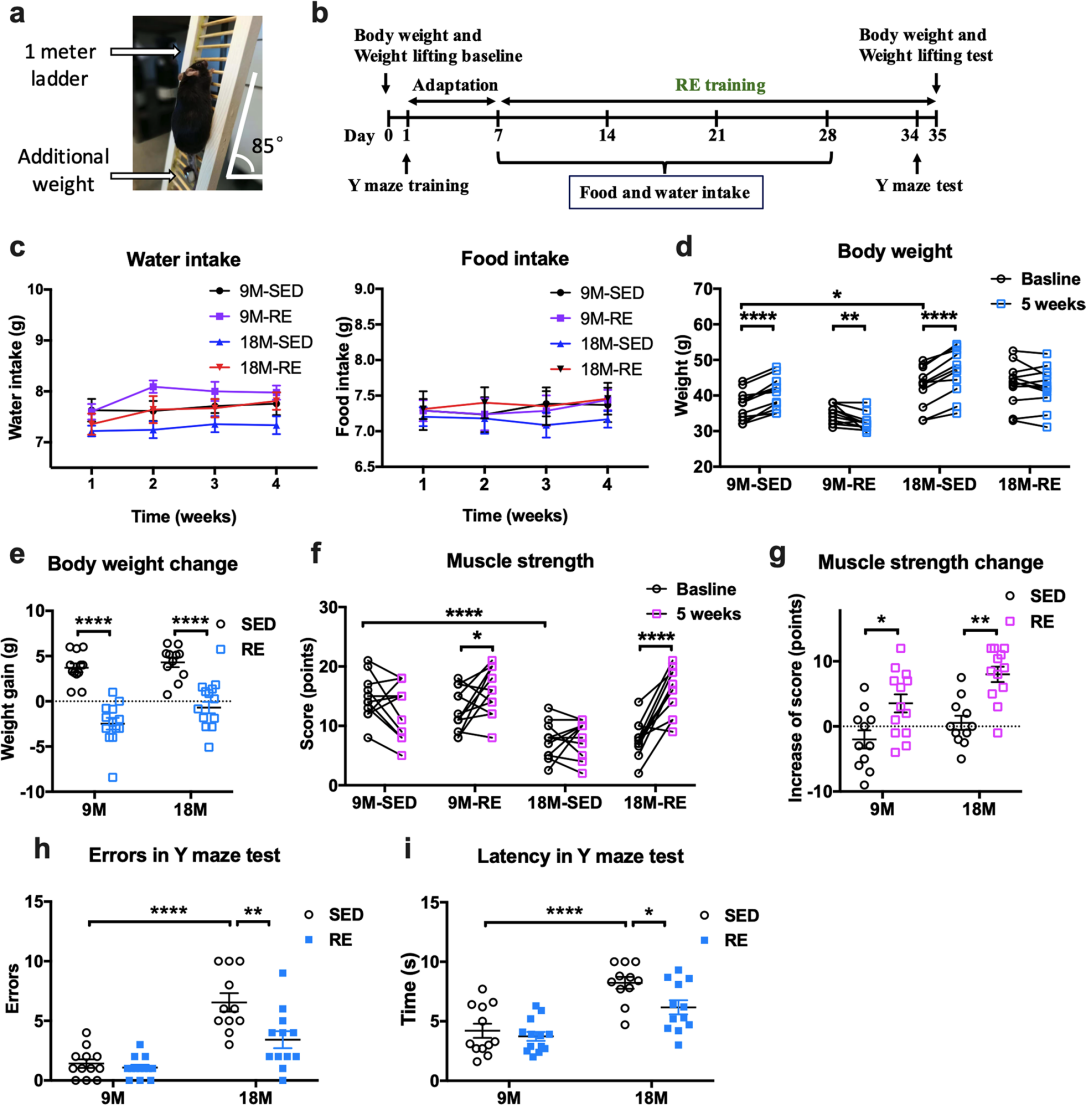
Resistance training enhanced the muscle strength and cognitive performance in 9-month and 18-month-old mice. **a** A picture of resistance exercise training apparatus. **b** A timeline of the experimental interventions. **c** The water and food intake during the training period. Data represented the averaged values of individual group and were analyzed by repeated measures of two-way ANOVA, followed by Bonferroni test as post hoc comparisons. **d** The body weight changes in mice from the four different groups in absolute values and **e** in grams gained or lost. **f** Muscle strength scores at baseline and after resistance exercise in absolute values and **g** in terms of points gained or lost. The score was calculated by the formula: [3 × (heaviest weight − 40 g) + (time held)]. Body weight and muscle strength values were analyzed by paired Student’s *t*-tests, paired with baseline. Body weight and muscle strength changes were analyzed by two-way ANOVA, followed by Tukey multiple comparisons test. **h** Number of errors in the Y-maze test after training. A higher number of errors indicate poorer performance. **i** Escape latency in the Y-maze test. A longer latency indicates poorer performance. These were analyzed by two-way ANOVA, followed by Tukey multiple comparisons test. *n* = 12 for 9M-SED, *n* = 13 for 9M-RE (except for muscle strength test, one mouse in middle-aged exercise group wounded its front paw on testing day, therefore its data about muscle strength was excluded), *n* = 11 for 18M-SED and *n* = 12 for 18M-RE. *SED* sedentary group, *RE* resistance exercise group. Data presented as mean ± SEM. *p < 0.05, **p < 0.01, ***p < 0.001, ****p < 0.0001

Mice were trained according to our previously published protocol using a 1-m ladder [20] (Fig. 1a and b). In brief, 1 week after familiarization with ladder climbing, the mice were trained on alternate days for 4 weeks with a 2 min rest between each climb. They were motivated to climb up 15 times per session, with progressively heavier weights attached to their tails. The weights were equivalent to 15%, 25%, 40% and 50% of their body weight, applied at 1, 2, 3 and 4 weeks, respectively. The intensity was carefully adjusted based on the individual performances at each exercise session.

The mice became familiarized with the ladder climb after around 3–4 secessions and those showing natural interest in this training protocol would climb up the ladder spontaneously. During experimentation, excessive stress to the mice was minimized by allowing them to first climb up the ladder spontaneously and only motivated them if they stopped in the middle, by a gentle touch to the tail (Additional file 1). Extra rest time was allowed for mice that displayed a refusal to climb or tachypnoea after their 2-min rest. If they still refused, the attached weight was progressively reduced by 5 g until they were willing to climb, then after were gradually reintroduced in subsequent climbs.

### Strength assessment

Muscle strength test was conducted following the protocol of Deacon et al. [21]. The mice were held by the middle of the tail and lowered to allow them to grasp a series of weights on the bench with all four paws. The duration of the weight being lifted clear of the table was timed and if it exceeds 3 s, the next heavier weight was tested. If the mouse drops the weight within 3 s, it was allowed to rest for 20 s and then reattempt with the same weight. Testing was completed if the mouse failed to hold the same weight after three consecutive attempts. A score was then calculated by the formula: [3 × (heaviest weight − 40 g) + (time held)].

### Anesthesia and surgery

A laparotomy was performed under sevoflurane anesthesia following our previous protocol taking approximately 30 min [22]. Using a rodent inhalation anesthesia apparatus (Harvard, US), anesthesia was induced with 5–6% sevoflurane (Sevorane TM, Abbott, Switzerland) in a perspex induction chamber, and maintained at 3–4% with 0.8 L/min oxygen flow. A 2.5-cm midline incision was made to enter the abdominal cavity. Approximately 10 cm of the intestine were exteriorized, rubbed vigorously for 1 min and were left exposed outside the abdominal cavity for a further 2 min before replacing it back into the abdominal cavity. Sterile chromic gut sutures (4-0, PS-2; Ethicon, USA) were used to suture the abdominal muscle and the skin in two separate layers. A heating pad was used to keep the mice warm, the rhythm and frequency of respiration, as well as the color of the paw, were monitored.

### Behavioral and cognitive assessment

The behavioral tests were performed from least-to-most stressful order to minimize possible interference by any residual distress from the previous test. They were conducted in a dedicated room with no natural light, noise, odor, or other animals not involved with testing. While still in their home cages the mice were acclimatized to this environment for 30 min. Before each round the experimental apparatus was cleaned using 70% ethanol to remove residual odors. The mice were tested in a random order between and within groups.

#### Open field test

The open field test makes use of rodents’ natural aversion to open spaces to assess general locomotor activity and anxiety of rodents [23] and was performed on postoperative day 12 using an enclosed gridded arena of 40 cm × 40 cm × 40 cm under dim lighting. The floor of the box was demarcated into 9 squares, each measuring approximately 13.3 cm × 13.3 cm, with the middle one designated as the central area. Each mouse was gently placed in that middle square and allowed to spontaneously explore the arena without any interference. This spontaneous activity of the mouse was video recorded for 10 min. The total exploration time in the central area indicates anxiety, whereas the frequency of crossing grid lines is a measure of locomotor activity.

A video tracking software, SMART 3.0 (Panlab SL) were used for data analysis following the operation manual. Specifically, the arena was equally divided into nine zones (as shown in Fig. 3b), the total exploration time of mice in the central area of 13.3 × 13.3 cm^2^ indicates anxiety/depression, and the total transition during different zones, total distance travelled and mean speed are measures of locomotor activity.

#### Novel object recognition test

The NOR test exploits a rodent’s preference to explore a novel rather than a familiar object to measure the animal’s recognition memory and exploration tendencies [24] and was performed 24 h after the open field test. Two identical objects (A + A) were placed at opposite corners in the arena and the mice were allowed to explore them freely for 10 min. Following a 6 h retention interval, the animals were placed back to the arena, this time with one object being the same (A) and the other being novel (B). We alternated the location of the novel versus familiar object for each batch of tests. An interaction was defined if the nose of the mouse points to the object within 2 cm. The discriminating index is the ratio of the time exploring the novel object over the total time spent exploring both objects.

#### Y-maze test

Y-maze test was performed following a previous published protocol [25]. The Y-maze test was designed to assess aversive memory and hippocampal-dependent spatial learning. The apparatus consists of three interconnected plastic compartments angled at 120° from each other to shape like the capital letter “Y”, with each compartment measuring 32 cm (long) × 10 cm (wide) × 10 cm (high). One of the compartments is white with a transparent cover, while the remaining two are black with an opaque cover. The floor of the black arms is lined with 3.2-mm stainless steel rods placed 8 mm apart capable of delivering electric shocks (2 Hz, 10 s, 40 ± 5 V). During the preoperative training, each mouse was allowed to explore the three arms freely for 5 min. Thereafter the animal was placed in the blind end of one of the black arms randomly. Electric shocks were applied until the animal enters the shock-free white compartment. A correct choice was recorded when the animal enters the white compartment within 10 s and stays there for at least 30 s. An error occurs when the animal enters the other black compartment or failed to enter and stay in the white compartment within the appropriate time. Successful training was defined as 9 consecutive correct choices. Postoperatively each mouse was tested 10 times and the number of errors and time before entering the shock-free compartment (latency) were recorded.

### Quantitative real-time polymerase chain reaction (PCR)

Under RNase-free conditions, the brain, muscle and liver tissues were separately homogenized using Tri-Reagent^®^ (MRC, USA). The RNA quality was assessed by optical density (OD) measurement (Nanodrop1000; Thermo Fisher Scientific, USA). Only isolated RNA samples with an OD 260/280 ratio > 1.8 and OD 260/230 ratio < 2.0 were used for analysis. Isolated RNA was further purified by removing genomic DNA with an Ambion^®^ DNA-freeTM DNA Removal Kit (Invitrogen, USA), then underwent reverse transcription using PrimeScript™ Master-Mix Kit (TAKARA, Japan). PCR was performed using StepOnePlus™ Real-Time PCR system (Applied Biosystems, USA) with the SYBR^®^ Premix Ex-TaqTM II Kit (TAKARA, Japan). The amplification conditions were 95 °C for 20 s, followed by 40 cycles of denaturation at 95 °C for 15 s, extension at different gene-specific annealing temperature as described in Additional file 2: Table S1 (which also included primer sequences), and data capture was conducted at 72 °C for 30 s. The relative levels of cytokines were normalized to those of the endogenous reference glyceraldehyde-3-phosphate dehydrogenase (GAPDH) following the 2^−ΔΔCt^ method.

### Milliplex cytokine assays

The protein levels of pro- and anti-inflammatory cytokine including IL-1β, IL-10, MCP-1, and TNF-α in the hippocampus were measured using a customized Milliplex Mouse Cytokine Immunoassay Kit (Millipore, 2620525) with Analyzer 3.1 Luminex 200 machine (Millipore, USA). Data were analyzed using corresponding software according to the manufacturer’s instructions.

### Western blot

Total lysates or cellular compartment fractions were prepared using RIPA buffer or commercial kit (procedure detailed in Additional file 2), subjected to 10%, 12.5%, or 15% polyacrylamide gels electrophoresis depending on the molecular weight of target protein, and transferred onto PVDF membranes. Non-specific binding sites were blocked with 5% non-fat milk for 1 h at room temperature. Then the membranes were incubated overnight at 4 °C with specific primary antibodies. After the incubation with horseradish peroxidase-conjugated secondary antibodies (DAKO, Denmark) for 2 h, the immunoreactive band signal intensity was subsequently visualized by chemiluminescence (WesternBright™ ECL, or WesternBright™ Quantum). Signals were captured by ChemiDoc™ Touch Imaging System (Bio-Rad, USA). Immunoblots were normalized for gel loading with β-actin or GAPDH antibodies (Sigma-Aldrich, USA). The intensities of chemiluminescent bands were measured by Image Lab™ Touch Software Version 1.2 (provided by Bio-Rad, USA). Primary and secondary antibodies used are shown in Additional file 2: Table S2.

### Immunofluorescent staining

Twenty-micron-thick coronal sections were cut for immunofluorescent staining following a previous protocol [26] (sections in 30 μm thickness were used for Iba-1 and GFAP staining. After antigen retrieval with 0.01 M citrate buffer (pH 6.0) at 90 °C for 15 min), 5% normal goat serum was used for blocking non-specific binding. The sections were then incubated with specific primary antibodies at 4 °C overnight. Sections were then incubated with specific Alexa Fluor 568 or 488 secondary antibodies (Invitrogen, USA) for 2 h at room temperature (Additional file 2: Table S2b). The counterstaining was performed with 3 μM 4′-6-diamidino-2-phenylindole (DAPI). Immunolabeled tissues were observed under a laser scanning confocal fluorescent microscope (Carl Zeiss LSM 700, Germany) equipped with ZEN light software at 1024 × 1024 resolution, Z-stack images were acquired. All quantitative analyses were performed on at least three images per animal from three independent experiments. The images from Iba-1 staining were analyzed by Image J similar to our previous protocol [27]. Firstly, the area of interest was selected in images of CA1, CA3 or DG in the hippocampus. Then, the threshold of the image was determined to highlight the cell body of microglial cells that labeled with Iba-1, by clicking ‘Analyze’ and then ‘Analyze particles’, the ImageJ gave the number and area of the selected dots. The number of dots represent microglia cell number, and cell body size is equal to area/number, the value was then normalized to control group [27]. We followed Tavares et al.’ methods [28] to perform the morphological analysis of astrocytes using Simple Neurite Tracer plugin of Image J. The investigator conducting data analysis was blinded to minimize analytical bias.

### Golgi staining

Whole brains were immediately removed without perfusion and rinsed with double distilled water for 2–3 s to remove any blood on its surface. Hito Golgi-Cox OptimStain Kit (Hitobiotec Inc., Wilmington, DE, USA) was used for the tissue preparation and staining procedure, conducted in strict adherence to the manufacturer’s user manual and material safety data sheet. A series of 150-μm-thick coronal sections were sliced from the hippocampus using a cryostat (Leica, Wetzlar, Germany). Pictures were taken of the hippocampal DG region of each brain. Dendritic branching was assessed using serial photographs taken with a 20× objective lens that provides sufficient dendritic branching resolution. Sholl analysis was conducted using a Sholl analysis plug-in (http://fiji.sc/Sholl-Analysis) for Image J software (National Institutes of Health, Bethesda, MD, USA) to demonstrate dendritic complexity.

Spine density was analyzed using serial photographs taken with 100× objective lens. Only neurons with well impregnated dendritic trees, intact cell bodies and dendrites in full view in the plane of section and not obscured by other blood vessels, astrocytes, or dendritic clusters were analyzed. Dendritic spine density of the DG was measured. For each cell, 20-μm-long segments of apical densities on the lower half of the apical segments were traced at 1000× magnification. The number of spines was recorded and together with the length of the dendritic segment the number of spines per μm was calculated. No corrections were made for spines obscured by the overlying dendrites, so the data obtained might be an underestimate of the actual density. The morphology analysis of neurons was performed by a blinded assessor.

### Transmission electron microscopy (TEM)

A Philips CM100 TEM was used to demonstrate the morphological changes in the mitochondria. Briefly, hippocampi were rapidly isolated and cut into 2-mm cubes followed by fixation in 2.5% glutaraldehyde (EM grade) in 0.1 M buffer overnight and then processed to slice by professional technicians in the Electron Microscope Unit of Queen Marry Hospital, the University of Hong Kong. To demonstrate mitochondria in the DG, the nucleus of DG granule cells was first identified under 700×, and photos with higher magnification focused on granule cells project zones were taken, which captured mitochondria both near the synapse and away from the nucleus. For each slice, 2200× and 3900× pictures were taken to analyze mitochondrial density and abnormalities, and ER–mitochondria contacts were analyzed using 11,500× pictures to demonstrate the ultrastructure. The subsequent number and morphology analysis of mitochondria was performed using Image J by a blinded assessor. The total number of mitochondria was manually determined using the “multi-point” tool, and abnormal mitochondria are then marked and calculated based on previously reported typical morphological change (abnormal mitochondria are characterized by irregular, swollen or whorling cristae, swollen outer or discontinuous inter or outer membranes [29], the typical morphology of abnormal mitochondria is shown in Fig. 7g). The ER–mitochondria contacts are measured using the “freehand line” tool to obtain the length of mitochondrial surface associated with the ER (< 30 nm), as well as the circumference of the mitochondria following the protocol of Stoica et al. [30].

### Statistical analysis

All data were first examined by the Shapiro–Wilk normality test using Prism 7.0 (Graphpad Software, USA) to check for normal distribution. A repeated-measures ANOVA was used to analyze the data obtained for the food and water intake. Either paired or unpaired two-tailed Student’s *t*-test was used to analyze the data obtained for body weight, muscle strength and RT-PCR. A two-way ANOVA was used for statistical analysis of parametric data for all the rest data, including behavioral test, Golgi staining, Immunofluorescent staining, western blot and TEM. All results were presented as the means ± standard error of the mean (SEM). In all cases, a *p*-value of less than 0.05 was considered statistically significant.

## Results

### General conditions and pre-existing cognitive impairment in aged mice improved with resistance exercise

Mice were trained using a 1-m ladder (Fig. 1a) and followed the testing and monitoring schedule as shown in Fig. 1b. Food and water intake did not differ between the active and sedentary groups (Fig. 1c). The mean baseline body weights for the sedentary and active group were, respectively, 37.17 ± 3.87 g versus 34.92 ± 2.30 g in the middle-aged mice and 42.39 ± 5.60 g versus 43.15 ± 5.66 g for the older mice. After 5 weeks of training, these values became 40.87 ± 4.27 g versus 32.45 ± 2.14 g and 46.70 ± 6.31 g versus 42.46 ± 5.43 g, respectively (Fig. 1d). The aged mice were heavier at baseline compared with their younger counterparts with the difference between 9M-SED versus 18M-SED being 5.23 ± 2.1 g (*p* = 0.0205) and between 9M-RE versus 18M-RE being 8.23 ± 1.78 g, (*p* = 0.0001 Fig. 1d). The body weights of sedentary mice of both age groups significantly increased after 5 weeks (3.71 ± 0.5 g, *p* < 0.0001 for 9M mice; 4.31 ± 0.5 g, *p* < 0.0001 for 18M mice, Fig. 1d). Resistance exercise reduced the body weight only in middle-aged but not the older mice (− 2.47 ± 0.65 g, *p* = 0.0024 for middle-aged mice; *p* = 0.3002 for aged mice, Fig. 1d) but prevented weight gain otherwise seen in sedentary aged mice (interaction: *F*_(1, 44)_ = 1.008, *p* = 0.3209; age factor: *F*_(1, 44)_ = 4.132, *p* = 0.0481; exercise factor: *F*_(1, 44)_ = 90.59, *p* < 0.0001. Multiple comparisons: − 5.0 ± 0.85 g, *p* < 0.0001 for aged mice, Fig. 1e).

Recent studies suggest muscle strength can be used as an index to monitor progression of cognitive decline in old adults [31]. Aged mice have significantly lower baseline muscle strength compared with their younger counterparts (− 7.27 ± 1.46 points, *p* < 0.0001, Fig. 1f). Five weeks of resistance training significantly improved the weight lifting performance from both groups (3.54 ± 1.39 points, *p* = 0.0256 for 9M mice; 7.99 ± 1.18 points, *p* < 0.0001 18M mice, Fig. 1f). A similar trend was seen in further analysis of muscle strength change (interaction: *F*_(1, 43)_ = 0.5549, *p* = 0.4604; age factor: *F*_(1, 43)_ = 7.468, *p* = 0.0091; exercise factor: *F*_(1, 43)_ = 25.71, *p* < 0.0001. Multiple comparisons: 5.538 ± 1.794, *p* = 0.0179 for 9M mice; 7.446 ± 1.828, *p* = 0.0011 for 18M mice, Fig. 1g). The effect of resistance exercise on learning and memory was evaluated by the Y-maze test. We found a significant interaction between age and exercise, suggesting that the effect of resistance exercise differed between middle-aged mice and aged mice (for errors, interaction: *F*_(1, 44)_ = 6.497, *p* = 0.0144; age factor: *F*_(1, 44)_ = 46.59, *p* < 0.0001; exercise factor: *F*_(1, 44)_ = 10.05, *p* = 0.0028, Fig. 1h. For latency, interaction: *F*_(1, 44)_ = 2.301, *p* = 0.1364, age factor: *F*_(1, 44)_ = 38.46, *p* < 0.0001; exercise factor: *F*_(1, 44)_ = 5.838, *p* = 0.0199, Fig. 1i). Multiple comparisons found that aged mice demonstrated deficits in aversive memory and hippocampal-dependent spatial learning (errors: 5.1 ± 0.79, *p* < 0.0001, Fig. 1h; latency: 4.03 ± 0.75 s, *p* < 0.0001, Fig. 1i) when compared with the middle-aged mice. Resistance exercise reduced both errors (− 3.1 ± 0.79, *p* = 0.0015, Fig. 1h) and latency (− 2.05 ± 0.75 s, *p* = 0.0439, Fig. 1i**)** in aged mice. These data suggested that the current training protocol prevented the aged mice from becoming overweight without affecting their food and water intake, improved their muscle strength and cognitive function.

### Resistance training improved postoperative cognitive performance in aged mice

**Fig. 2:**
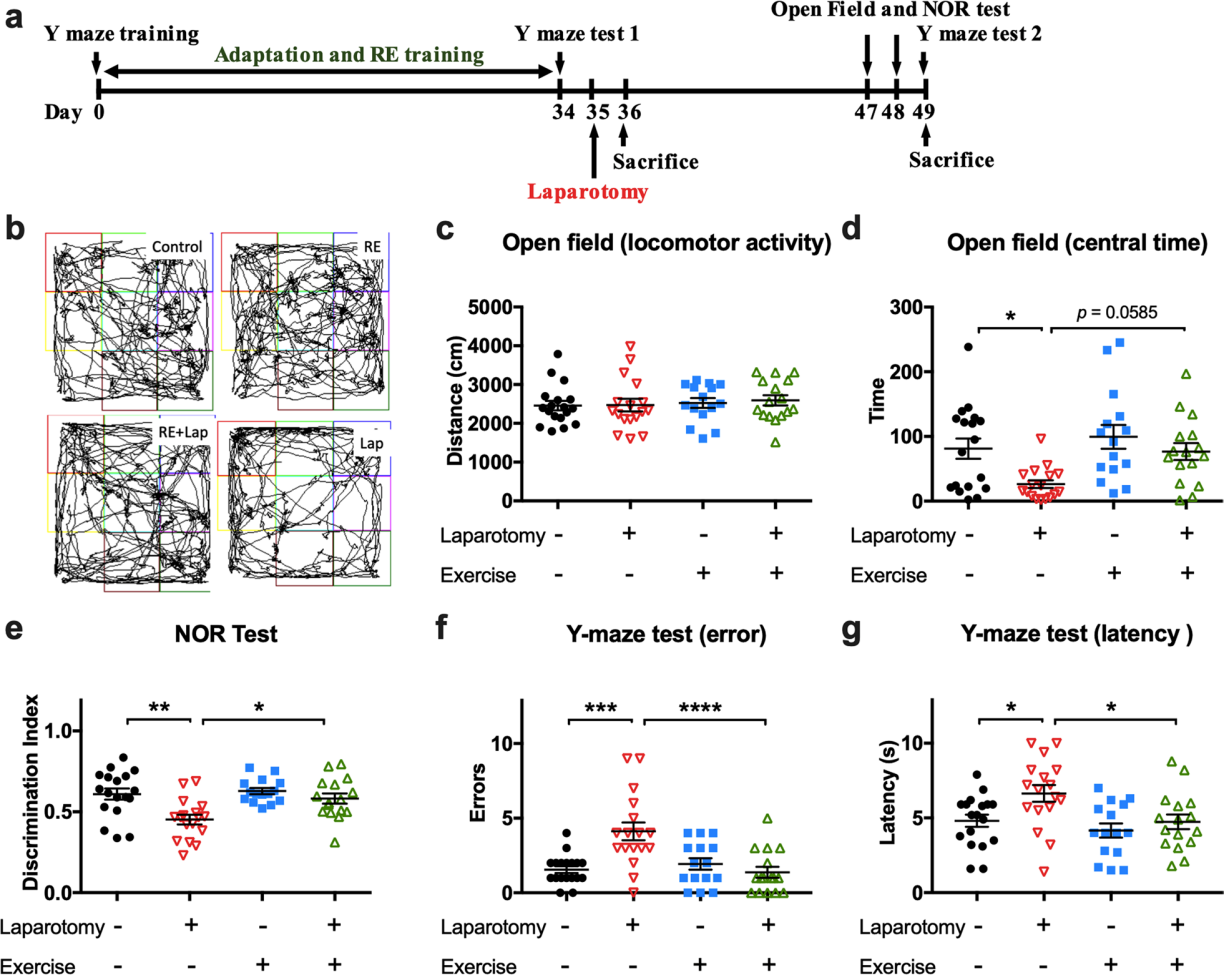
Resistance training alleviated laparotomy-induced cognitive dysfunction in aged mice. **a** Experimental workflow showing the time points of resistance exercise training, surgery and behavioral tests. **b** Representative moving traces of mice in the open field arena. **c** Total distance accumulated during testing by each mouse. A reduction would indicate impaired locomotion compared with their counterparts. **d** Time of central duration in open field test. Reduced time spent in the central square indicates the anxiety in the rodent. Data were analyzed by two-way ANOVA, followed by Tukey multiple comparisons test. **e** Recognition index from novel object recognition test (Time^new^/Time^old^). A significant higher index was observed in laparotomy group with RE training compared with laparotomy alone group indicating better recognition memory in the former group. **f** Escape latency and **g** number of errors in the Y-maze test. Significant decrease of error number and escape latency was found in the RE + Lap compared with Lap group. Data were analyzed by two-way ANOVA, followed by Tukey multiple comparisons test. *n* = 18 for control, *n* = 15 for resistance exercise (RE) group, 16 for resistance exercise before laparotomy (RE + Lap) group and *n* = 17 for laparotomy (Lap) group. Data presented as mean ± SEM. *p < 0.05, **p < 0.01, ***p < 0.001, ****p < 0.0001

A laparotomy was performed around 24 h after the last resistance training session. Open field and NOR tests were conducted on postoperative days (PODs) 12 and 13 (Fig. 2a). As can be seen from Fig. 2b and c, locomotor activity was not affected in any of the groups. Surgery induced anxiety in the laparotomy group as indicated by a decreased central duration compared with control (interaction: *F*_(1, 62)_ = 1.347, *p* = 0.2502; laparotomy factor: *F*_(1, 62)_ = 7.808, *p* = 0.0069; exercise factors: *F*_(1, 62)_ = 6.119, *p* = 0.0161. Multiple comparisons: − 54.93 ± 19.04 s; *p* = 0.0268 compared to control, Fig. 2d). Pre-operative resistance exercise appears to exert some anxiolytic effects, but the change in central duration time in the RE + Lap group failed to reach statistical significance (*p* = 0.0585 compared to laparotomy group, Fig. 2d).

Recognition memory deficits was observed in the post-surgical mice as indicated by the decrease in the discrimination index as measured by the NOR test (interaction: *F*_(1, 62)_ = 3.449, *p* = 0.0680; laparotomy factor: *F*_(1, 62)_ = 11.6, *p* = 0.0012; exercise factor: *F*_(1, 62)_ = 6.158, *p* = 0.0158. Multiple comparisons: − 0.16 ± 0.04, *p* = 0.0016 compared to control, Fig. 2e). Similarly, in the Y-maze test, more error numbers (interaction: *F*_(1, 62)_ = 14.01, *p* = 0.0004; laparotomy factor: *F*_(1, 62)_ = 5.778, *p* = 0.0192; exercise factor: *F*_(1, 62)_ = 8.049, *p* = 0.0061. Multiple comparisons: 2.56 ± 0.57, *p* = 0.0002, Fig. 2f) and longer time of escape latency (interaction: *F*_(1, 62)_ = 1.65, *p* = 0.2037; laparotomy factor: *F*_(1, 62)_ = 6.092, *p* = 0.0164; exercise factor: *F*_(1, 62)_ = 6.846, *p* = 0.0111. Multiple comparisons: 1.83 ± 0.67 s, *p* = 0.0392, Fig. 2g) were found in laparotomy group compared with control. On the other hand, preoperative resistance training attenuated these changes when compared to laparotomy only mice (discrimination index: 0.13 ± 0.04, *p* = 0.0162; errors: − 2.7 ± 0.6, *p* < 0.0001; latency: − 1.9 ± 0.7 s, *p* = 0.0368, Fig. 2e–g). These data indicated that laparotomy induced postoperative cognitive dysfunction, while preoperative resistance training ameliorated this cognitive decline in the aged mice.

### Resistance training attenuated the loss of dendritic process complexity and spine density following laparotomy

**Fig. 3:**
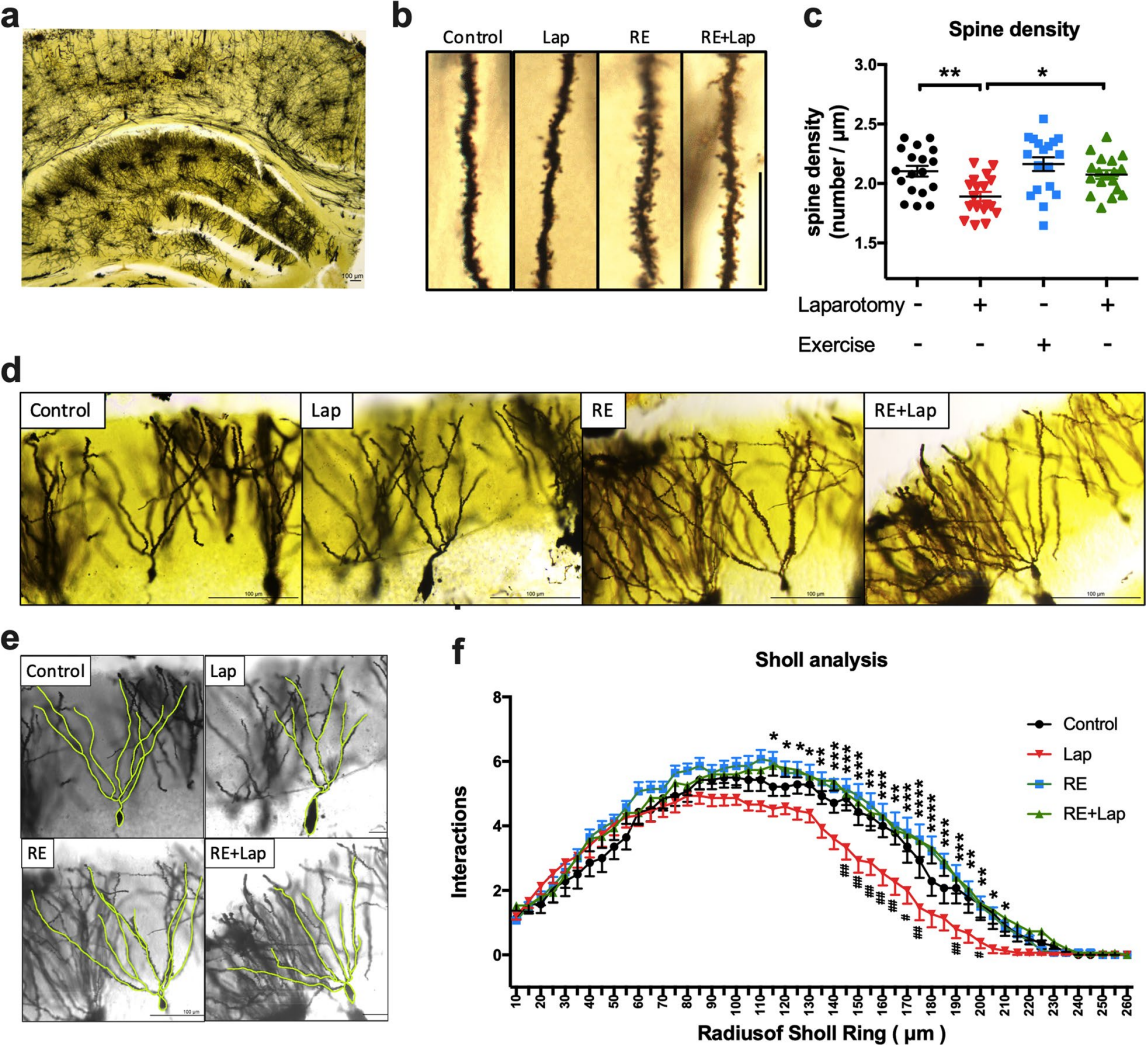
Resistance training attenuated the synaptic deficiency in aged mice following surgery. **a** A representative photograph of Golgi staining of the hippocampus. **b** Representative pictures of dendritic spines for the four experimental groups. The spine density is significantly decreased in the lap compared with control. **c** Quantitative analysis of spine density for the four experimental groups. An increase in spine density was observed in the laparotomy group with RE training compared with laparotomy only group. Data were analyzed by two-way ANOVA, followed by Tukey multiple comparisons test, *n* = 18 in total, 4–5 dendrites were chosen at random from 4 mice in each group. **d**–**f** Sholl profiles generated with semi-automated analyses revealing process complexity of dendrites. Significant increases in branch points 120 μm proximal to the cell body layer from laparotomy group with RE training when compared with laparotomy group in areas. Data were analyzed by repeated-measures two-way ANOVA, followed by Bonferroni test as post hoc comparisons, *n* = 14–15 in total, 3–4 neurons were chosen from 4 mice in each group. Data were presented as mean ± SEM, *p < 0.05, **p < 0.01 and ***p < 0.001, resistance exercise before laparotomy (RE + Lap) versus laparotomy (Lap) group. ^#^*p* < 0.05 and ^##^*p* < 0.01 Control versus laparotomy (Lap)

To evaluate whether the beneficial effect of resistance exercise on postoperative cognitive function was accompanied by structural improvement in neurons, we used Golgi staining to delineate the morphology of dendrites and spines (Fig. 3a and b). The spine density was significantly decreased in the laparotomy group compared with control (interaction: *F*_(1, 68)_ = 1.89, *p* = 0.1738; laparotomy factor: *F*_(1, 68)_ = 10.96, *p* = 0.0015; exercise factor: *F*_(1, 68)_ = 7.189, *p* = 0.0092. Multiple comparisons: − 10.63 ± 3.21%, *p* = 0.0079, Fig. 3c), while preoperative resistance training ameliorated this reduction in postoperative mice (9.21 ± 3.21%, *p* = 0.0275, Fig. 3c). Sholl profiles generated by semi-automated analysis showed a reduction in dendritic crossings following surgery, indicative of decreased dendritic length and branching within 145–200 μm of the radius (interaction: *F*_(150, 2700)_ = 1.833, *p* < 0.0001; laparotomy factor: *F*_(50, 2700)_ = 194.5, *p* < 0.0001; exercise factor: *F*_(3, 54)_ = 7.736, *p* = 0.0002. Multiple comparisons: *p* < 0.05, *p* < 0.01, compared to control, Fig. 3d–f). The resistance exercise group had greater numbers of dendritic crossings within 115–210 μm of the radius compared to laparotomy only group (*p* < 0.05, *p* < 0.01, *p* < 0.001, *p* < 0.0001, compared to laparotomy group, Fig. 3d–f). These results indicated that preoperative resistance training could alleviate surgery-induced dendritic and synaptic deficit in postoperative mice.

### Preoperative pro-inflammatory disposition in aged mice and early inflammatory responses were reduced by resistance exercise

**Fig. 4:**
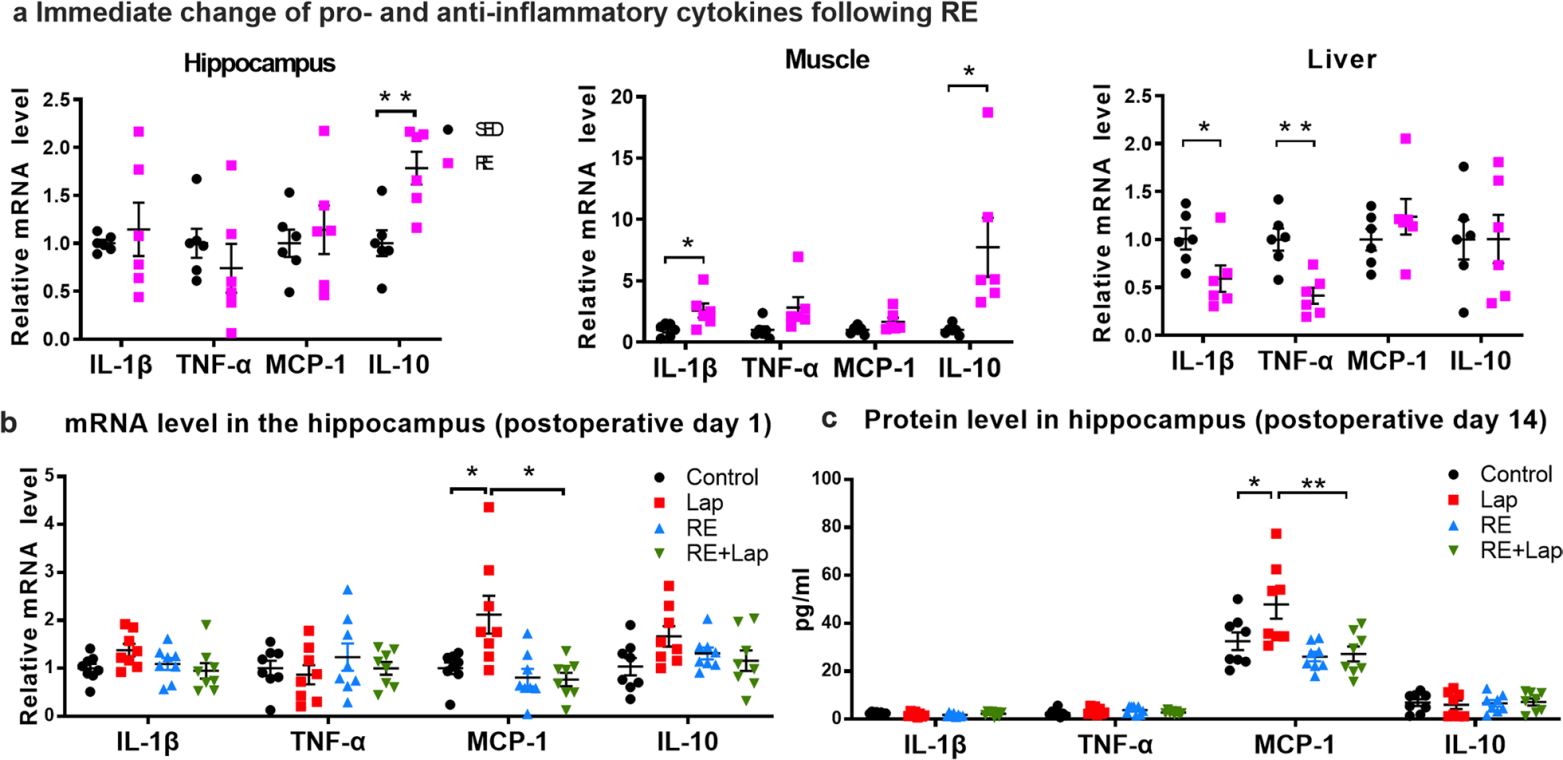
Resistance training inhibited inflammatory responses in the brain and peripheral organs of aged mice following surgery. **a** Relative mRNA levels of the inflammatory cytokines IL-1β, TNF-α, MCP-1 and the anti-inflammatory IL-10 in the hippocampus, muscle and liver after RE training. Data analyzed by unpaired Student’s *t*-test, *n* = 8. Data presented as mean ± SEM. *p < 0.05, **p < 0.01. **b** Relative mRNA levels of IL-1β, TNF-α, MCP-1 and IL-10 in the hippocampus at 24 h after surgery. Significant reduction in MCP-1 expression was observed in RE + Lap group. **c** Protein levels of IL-1β, TNF-α, MCP-1 and IL-10 in the hippocampus at 14 days after surgery. Significant reduction in MCP-1 expression was also observed in RE + Lap group. Data analyzed by one-way ANOVA with Tukey post hoc test, *n* = 8. Data presented as mean ± SEM. *p < 0.05

Both animal and human data suggest that PNDs are associated with chronic neuroinflammation following surgery and causing neural dysfunction [9]. The acute effect of resistance exercise on peripheral and central inflammatory cytokines were first analyzed in hippocampal, muscle and liver tissues obtained 30 min after the last resistance training session. Differential changes in the expression of cytokines were demonstrated in the hippocampus (up-regulation in IL-10: 0.78 ± 0.22, *p* = 0.0048; Fig. 4a), muscle (up-regulation in IL-1β: 1.58 ± 0.61, *p* = 0.0276; up-regulation in IL-10: 6.7 ± 2.4, *p* = 0.0193; Fig. 4a) and liver (down-regulation in IL-1β: − 0.42 ± 0.18, *p* = 0.0406; down-regulation in TNF-α: − 0.59 ± 0.14, *p* = 0.0022; Fig. 4a), with no changes seen in the expression of MCP-1 at this time point. With a reduction in pro-inflammatory cytokines in the liver and the elevated levels of anti-inflammatory IL-10 in the hippocampus and muscle, these data demonstrated that resistance training may exert anti-inflammatory effects in the periphery and the CNS.

For the anti-inflammatory effect of resistance training, we observed a significant increase in the mRNA expression of MCP-1 in the hippocampus of postoperative mice compared to that of control at 24 h after surgery (Interaction: *F*_(1, 28)_ = 4.531, *p* = 0.0422; laparotomy factor: *F*_(1, 28)_ = 5.756, *p* = 0.0233; exercise factor: *F*_(1, 28)_ = 6.207, *p* = 0.0189. Multiple comparisons: 1.16 ± 0.36, *p* = 0.017, Fig. 4b). Preoperative resistance exercise reduced the expression of MCP-1 in the hippocampus in postoperative mice (− 1.18 ± 0.36, *p* = 0.014, Fig. 4b). Consistently, a significant increasing in the protein level of MCP-1 was demonstrated in laparotomy group 14 days following surgery (Interaction: *F*_(1, 28)_ = 3.288, *p* = 0.0805; laparotomy factor: *F*_(1, 28)_ = 4.367, *p* = 0.0458; exercise factor: *F*_(1, 28)_ = 11.78, *p* = 0.0019. Multiple comparisons: 15.38 ± 5.57, *p* = 0.0468, Fig. 4c), which was also reduced by preoperative resistance exercise (− 20.67 ± 5.57, *p* = 0.0048, Fig. 4c). Surprisingly, no significant difference was observed in other cytokines including IL-1β, TNF-α and IL-10 among all groups (Fig. 4b).

### Resistance exercise alleviated prolonged inflammatory response following surgery

**Fig. 5:**
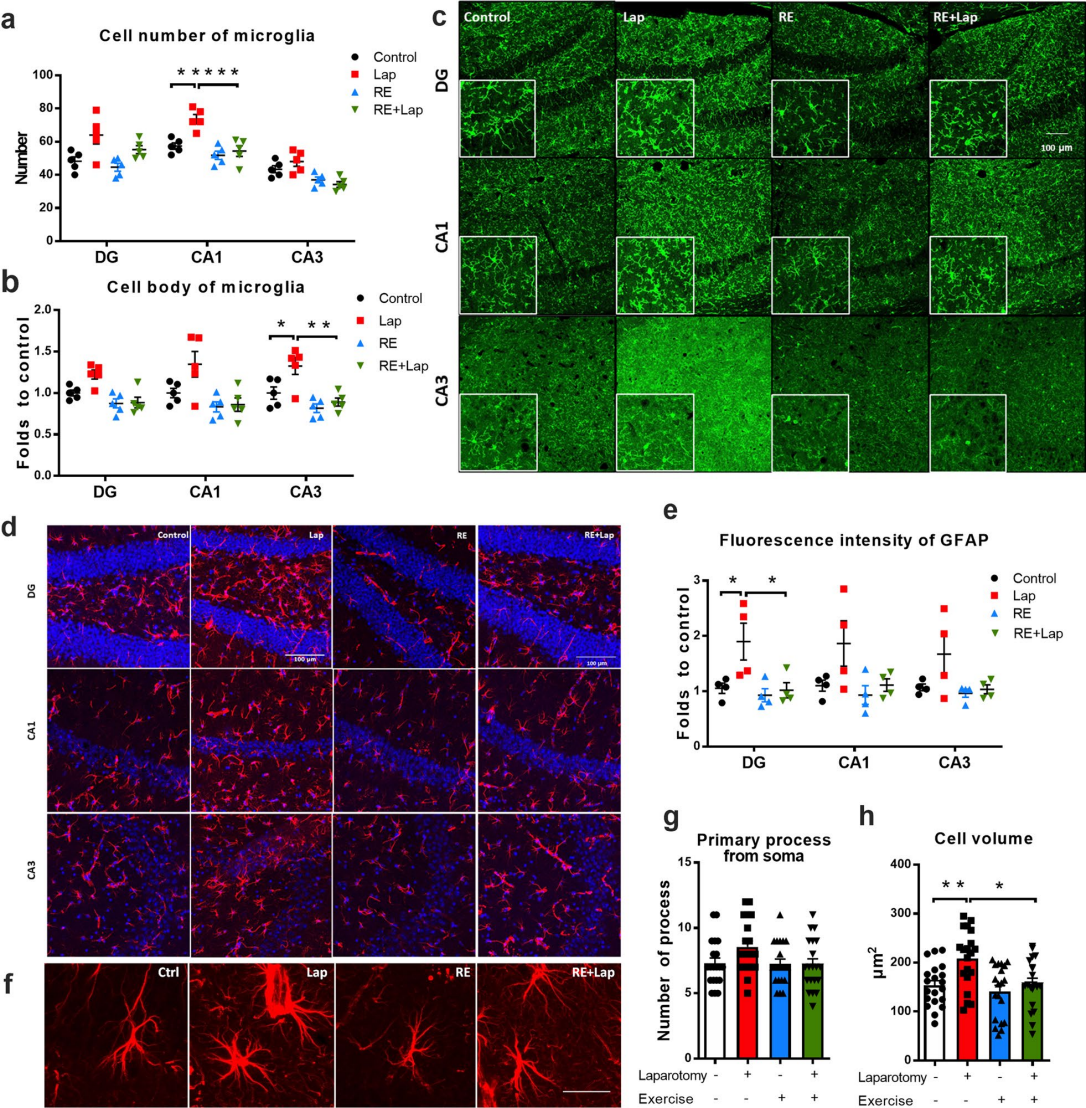
Resistance training ameliorated prolonged activation of astroglial in the hippocampus following surgery. **a** Iba-1^+^ microglia and **b** Iba-1^+^ microglia body size. **c** Representative z-stacked confocal images showing Iba-1^+^ microglia (green) in the DG, CA1 and CA3 sub-regions of the hippocampus; Scale bar: 100 μm. Significant reduction in Iba-1 + cells and morphological changes were observed in the RE + Lap compared with Lap group. **d** Representative z-stacked confocal images showing GFAP^+^ astrocytes (red) in the DG, CA1 and CA3 sub-regions of the hippocampus. Scale bar = 100 μm. **e** Quantitative analysis of the fluorescence intensity of GFAP ^+^ astrocytes in the different hippocampal regions using Image J. Significant reduction in GFAP^+^ immunofluorescence was observed in RE + Lap compared with Lapgroup. **f** Representative enlarged confocal images of GFAP^+^ astrocytes. Scale bar: 20 μm. **g** Count of primary processes from cell bodies of GFAP^+^ astrocyte. **h** GFAP^+^ astrocyte cell volume in different experimental groups. Significant reduction in GFAP^+^ cells volume was observed in the RE + Lap group. Data analyzed by two-way ANOVA, followed by Tukey multiple comparisons test, *n* = 8 for RT-PCR, *n* = 5 for IBA1 staining, *n* = 4 for GFAP staining, *n* = 20 for morphological analysis of astrocytes. Data presented as mean ± SEM. *p < 0.05, **p < 0.01, ***p < 0.001

The sustained effect of resistance exercise on postoperative inflammation was examined on postoperative day 14. Microglia play a key role in the neuroinflammatory response. An increase in the number of Iba-1 label positive microglia was found in the CA1 region of the hippocampus compared to control (interaction: *F*_(1, 16)_ = 6.578, *p* = 0.0208; laparotomy factor: *F*_(1, 16)_ = 12.57, *p* = 0.0027; exercise factor: *F*_(1, 16)_ = 21.87, *p* = 0.0003. Multiple comparisons: 16.2 ± 3.8, *p* = 0.0032; Fig. 5a). Activated microglia with hypertrophic cell body were also observed in the CA3 region of the hippocampus compared to control (interaction: *F*_(1, 16)_ = 3.053, *p* = 0.0997; laparotomy factor: *F*_(1, 16)_ = 7.591, *p* = 0.0141; exercise factor: *F*_(1, 16)_ = 18.23, *p* = 0.0006. Multiple comparisons: CA3: 0.33 ± 0.10, *p* = 0.0266, Fig. 5b). Preoperative resistance training attenuated this microglial activation, which was reflected by the reduction of Iba-1 positive cells (CA1: − 19.2 ± 3.8, *p* = 0.0005, Fig. 5a), as well as the cell body size (CA3: − 0.44 ± 0.10, *p* = 0.0031, Fig. 5b) compared to the laparotomy only group.

Astrocytes mediate microglial activation in surgery-induced neuroinflammation through different intracellular signaling [32]. We showed notable astrogliosis in the DG region following surgery, with an increase of GFAP intensity compared to control (interaction: *F*_(1, 12)_ = 3.753, *p* = 0.0766; laparotomy factor: *F*_(1, 12)_ = 5.755, *p* = 0.0336; exercise factor: *F*_(1, 12)_ = 6.664, *p* = 0.0240. Multiple comparisons: 0.84 ± 0.28, *p* = 0.043, Fig. 5e) and cell volume (interaction: *F*_(1, 76)_ = 2.832, *p* = 0.0965; laparotomy factor: *F*_(1, 76)_ = 9.053, *p* = 0.0036; exercise factor: *F*_(1, 76)_ = 7.513, *p* = 0.0076. Multiple comparisons: 54.6 ± 16.5, *p* = 0.0075; Fig. 5h). Preoperative resistance exercise ameliorated these changes compared to laparotomy only group (GFAP intensity: − 0.88 ± 0.28, *p* = 0.034; cell volume: − 51.58 ± 16.46, *p* = 0.013, Fig. 5d–h). These findings suggested that laparotomy triggered both microglial and astrocytic activation in the hippocampus, while preoperative resistance training alleviated these conditions in the postoperative mice.

### Alterations in myokines and intracellular signal pathways following resistance exercise and surgery

**Fig. 6:**
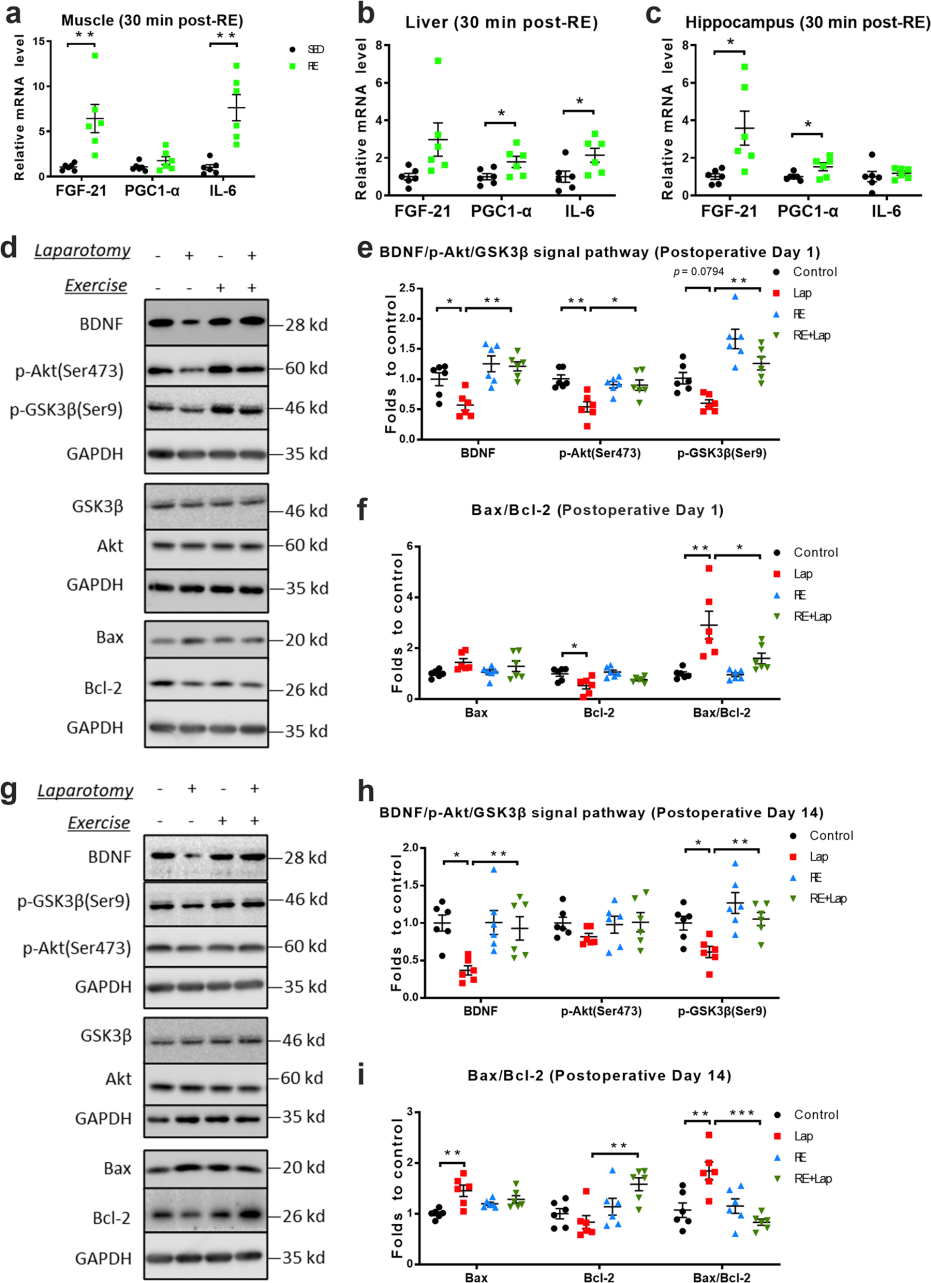
Resistance training modulated different myokines and intracellular signal pathways in the brain following surgery. **a**–**c** Relative mRNA levels of FGF-21, PGC-1α and IL-6 in the hippocampus, muscle and liver. **d**–**f** Representative blots and quantitative analysis of key proteins in intracellular signal transduction in the hippocampus on postoperative day 1 and (**g**–**i**) day 14, respectively. The intensity of band of BDNF, Akt, GSK3β, Bax and Bcl-2 was normalized to GAPDH. Data were analyzed by two-way ANOVA, followed by Tukey multiple comparisons test, *n* = 6. Data presented as mean ± SEM, *p < 0.05, **p < 0.01, ***p < 0.001

Various forms of exercise, particularly of the aerobic type, have been shown to trigger production of myokines that can reduce neuroinflammation [15] and confer positive effect in cognition [33]. PGC-1α is an important regulator of mitochondrial biogenesis, and it is known that Bcl-2 and Bax regulate the mitochondria-related intrinsic apoptosis by regulating the permeabilization of mitochondrial membrane, cytochrome *c* release and mitochondrial function [34]. The acute effect of resistance exercise on myokines were analyzed by using tissues obtained 30 min after the last resistance training trail. The data showed that resistance training increased levels of myokines as measured by RT-PCR in the muscle (up-regulation in FGF-21: 5.38 ± 1.57, *p* = 0.0066; up-regulation in IL-6: 6.62 ± 1.49, *p* = 0.0012; Fig. 6a), liver (up-regulation in PGC1-α: 0.788 ± 0.3371, *p* = 0.0415; IL-6: 1.141 ± 0.4779, *p* = 0.0381; Fig. 6b), and hippocampus (up-regulation in FGF-21: 2.58 ± 0.92, *p* = 0.0188; up-regulation in PGC1-α: 0.52 ± 0.22, *p* = 0.0405; Fig. 6c).

Previous reports have demonstrated the relationship between exercise, myokines and hippocampal BDNF signaling [35]. The expression of BDNF/Akt/GSK3β and Bax/Bcl-2 were evaluated at 24 h and 14 days after surgery, respectively. On postoperative day 1, a mild decrease in BDNF (interaction: *F*_(1, 20)_ = 3.59, *p* = 0.0727; laparotomy factor: *F*_(1, 20)_ = 5.325, *p* = 0.0318; exercise factor: *F*_(1, 20)_ = 19.31, *p* = 0.0003. Multiple comparisons: − 0.408 ± 0.144, *p* = 0.035, Fig. 6e) and significant decrease in phospho-Akt at serine 473 in the laparotomy group were observed when compared to control (interaction: *F*_(1, 20)_ = 9.614, *p* = 0.0056; laparotomy factor: *F*_(1, 20)_ = 10.15, *p* = 0.0046; exercise factor: *F*_(1, 20)_ = 3.137, *p* = 0.0918. Multiple comparisons: − 0.463 ± 0.104, *p* = 0.0013, Fig. 6e). Laparotomy also increased the Bax/Bcl-2 ratio compared to control (interaction: *F*_(1, 20)_ = 4.66, *p* = 0.0432, laparotomy factor: *F*_(1, 20)_ = 18.44, *p* = 0.0004; exercise factor: *F*_(1, 20)_ = 5.131, *p* = 0.0348. Multiple comparisons: − 1.914 ± 0.42, *p* = 0.001, Fig. 6f). On the other hand, preoperative resistance training attenuated these changes compared to the laparotomy group (BDNF: 0.641 ± 0.144, *p* = 0.001; p-Akt: 0.359 ± 0.104, *p* = 0.0126, p-GSK3β: 0.6764 ± 0.1613, *p* = 0.0023; Bax/Bcl-2 ratio: − 1.312 ± 0.42, *p* = 0.021, Fig. 6d–f).

On postoperative day 14, different changes were observed in these markers compared with postoperative day 1. After laparotomy, a significant decrease was observed in BDNF (interaction: *F*_(1, 20)_ = 4.673, *p* = 0.0429; *F*_(1, 20)_ = 7.709, *p* = 0.0116; *F*_(1, 20)_ = 4.937, *p* = 0.0380. Multiple comparisons: − 0.6325 ± 0.1811, *p* = 0.0113, Fig. 6h) and phospho-GSK3β at serine 9 (interaction: *F*_(1, 20)_ = 1.229, *p* = 0.2808; laparotomy factor: *F*_(1, 20)_ = 9.424, *p* = 0.0060; exercise factor: *F*_(1, 20)_ = 16.06, *p* = 0.0007. Multiple comparisons: 0.387 ± 0.131, *p* = 0.0362, Fig, 6h). The Bax/Bcl-2 ratio was also increased, which was similar to that on postoperative day 1 (*F*_(1, 20)_ = 15.83, *p* = 0.0007; laparotomy factor: *F*_(1, 20)_ = 2.735, *p* = 0.1138; exercise factor: *F*_(1, 20)_ = 11.5, *p* = 0.0029. Multiple comparisons: 0.774 ± 0.194, *p* = 0.004, Fig. 6i). However, no significant modulation of phosphorylated Akt was seen in the laparotomy group. On the other hand, prior resistance exercise restored these changes to that comparable to the control (BDNF: 0.562 ± 0.181, *p* = 0.027; phospho-GSK3β at serine 9: 0.4739 ± 0.131, *p* = 0.0086; Bax/Bcl-2 ratio: 1.012 ± 0.1943, *p* = 0.0002, Fig. 6g–i). Taken together, these results suggested the possibility that preoperative resistance training may ameliorate laparotomy-induced cognitive decline through inducing myokines and the subsequent BDNF/Akt signaling in the hippocampus of aged mice.

### Resistance exercise restored surgery-induced deficits in mitochondria density and morphology in the hippocampus

**Fig. 7:**
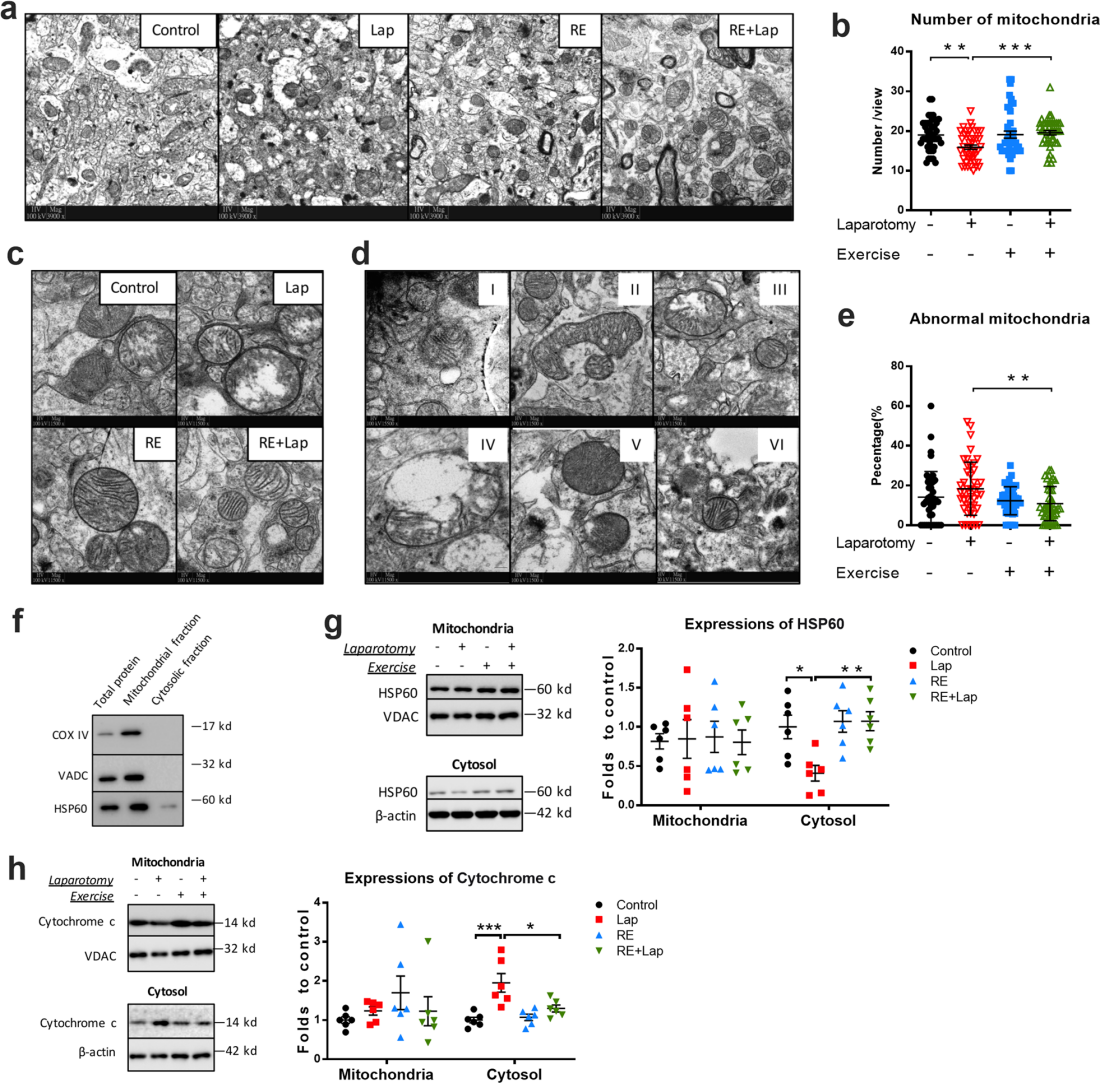
Resistance training improved mitochondrial density, morphology and protein expression following surgery. **a** Quality control of mitochondrial fraction isolation, equivalent amounts (10 µg) of total protein, mitochondrial and cytosolic fractions were analyzed for mitochondrial enriched markers as indicated. **b** Representative blots and quantitative analysis of HSP60 in mitochondrial and cytosolic fractions, respectively. **c** Representative blots and quantitative analysis of cytochrome *c* in mitochondrial and cytosolic fractions, respectively. The intensity of band was normalized to that of VDAC (mitochondrial fractions) or β-actin (cytosolic fractions). **d** Representative TEM pictures of mitochondria. **e** Quantitative analysis of mitochondrial density. **f** and **g** Representative TEM pictures of abnormal mitochondria, including type I whirling cristae; type II swollen cristae; type III deficient cristae; type IV swollen outer membrane; type V sub-membrane vesicle and type VI discontinuous inter/outer membrane. **h** Quantification of mitochondrial structural defects. Abnormal mitochondria with type I–VI have been expressed a percentage of the total pool of mitochondria. Data analyzed by two-way ANOVA, followed by Tukey multiple comparisons, for western blot: *n* = 6; for TEM: *n* = 44 comprised 11 pictures from 4 mice in each group. Data are shown as a mean ± SEM. *p < 0.05, **p < 0.01, ***p < 0.001, ****p < 0.0001

PGC-1α is a master regulator of mitochondrial biogenesis and the PGC-1α/BDNF pathway is closely involved with mitochondrial biogenesis and dynamic, which play important roles in the formation and maintenance of hippocampal dendritic spines and synapses [36]. We next explored the effects of resistance training on mitochondrial health. Transmission electron microscopy (TEM) demonstrated a decreased in the total number of mitochondria after laparotomy (interaction: *F*_(1, 172)_ = 7.266, *p* = 0.0077; laparotomy factor: *F*_(1, 172)_ = 3.88, *p* = 0.0505; exercise factor: *F*_(1, 172)_ = 8.03, *p* = 0.0052. Multiple comparisons: − 0.3068 ± 0.9301, *p* = 0.0064; Fig. 7a and b), which was not seen in the RE + Lap group (RE + Lap compared to Lap: 0.3636 ± 0.9301, *p* = 0.0008; Fig. 7a and b). Although there was no significant difference between the control and surgery groups, resistance exercise significantly reduced the percentage of abnormal mitochondria (interaction: *F*_(1, 172)_ = 3.039, *p* = 0.0831; laparotomy factor: *F*_(1, 172)_ = 0.7192, *p* = 0.3976; exercise factor: *F*_(1, 172)_ = 8.117, *p* = 0.0049. Multiple comparisons: − 0.7502 ± 0.023%, *p* = 0.0076; Fig. 7c–e).

Mitochondrial and cytoplasmic proteins were isolated using commercial reagents, the isolating efficacy of the reagent is reliable as the marker of mitochondria including COXIV and VADC are exclusively expressed in mitochondrial factions, while HSP60, a mitochondrial matrix localized protein that can also expressed in the cytoplasm, is found to be detected both in mitochondrial and cytosolic fractions (Fig. 7f). Our data demonstrated a significant decrease in HSP60 in the cytosolic fraction from the laparotomy group (interaction: *F*_(1, 20)_ = 5.291, *p* = 0.0323; laparotomy factor: *F*_(1, 20)_ = 5.194, *p* = 0.0338; exercise factor: *F*_(1, 20)_ = 8.106, *p* = 0.010. Multiple comparisons: − 0.5908 ± 0.1825, *p* = 0.0198, Fig. 7g). Importantly, an increase in cytochrome *c* was seen in the cytosolic fraction following surgery (interaction: *F*_(1, 20)_ = 6.955, *p* = 0.0158; laparotomy factor: *F*_(1, 20)_ = 18.45, *p* = 0.0004; exercise factor: *F*_(1, 20)_ = 4.514, *p* = 0.0463. Multiple comparisons: 0.951 ± 0.194, *p* = 0.0005; Fig. 7h). Both of these changes were reduced by prior resistance exercise (RE + Lap compared to Lap, HSP60: − 0.6641 ± 0.1825, *p* = 0.0082; cytochrome *c*: − 0.6533 ± 0.194, *p* = 0.0149, Fig. 7g and h). There was no significant change in relative levels of HSP60 or cytochrome *C* in the mitochondria fractions (Fig. 7g and h). These data implicated that laparotomy induced mitochondrial deficit and depletion in the hippocampus, while preoperative resistance training reversed this mitochondrial deficit in the aged mice.

### Modulatory effects of resistance exercise and surgery on mitochondria dynamics and synaptic markers in the hippocampus

**Fig. 8:**
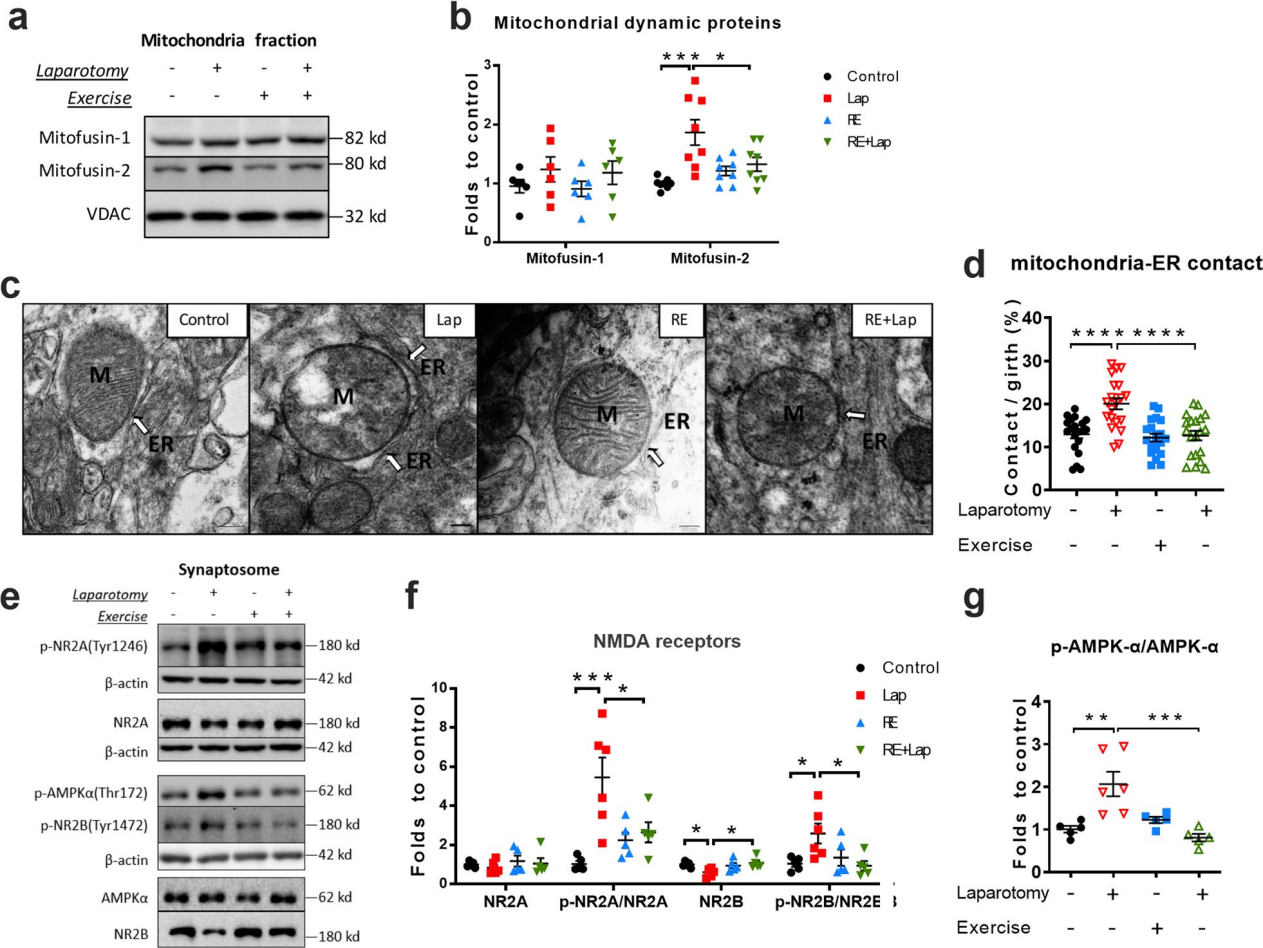
Resistance training modulated mitochondria dynamic following surgery. **a** and **b** Representative blots and quantitative analysis of mitofusin-1 and mitofusin-2 in the mitochondria fraction, the intensity of band was normalized to that of VDAC. *n* = 6 for mitofusin-1, 8 for mitofusin-2. **c** Representative TEM pictures of ER–mitochondria contacts as indicated by the white arrow. **d** Quantification of ER–mitochondria contacts, demonstrating by the contact area divided by the mitochondrial girth. *n* = 20 comprised 5 pictures from 4 mice in each group. **e**–**g** Representative blots and quantitative analysis of NMDARs and AMPK-α, with the intensity of band normalized to that of β-actin. Data analyzed by two-way ANOVA, followed by Tukey multiple comparisons test, *n* = 5 (except for Lap group, *n* = 6 for the expression of proteins in synaptosome). Data presented as mean ± SEM, *p < 0.05, **p < 0.01, ***p < 0.001. M: mitochondria; ER: endoplasmic reticulum

Mitochondria undergo ‘mitochondrial dynamics’, which are critical for various cellular processes including inflammation, apoptosis, astrogliosis and mitochondrial quality control [37]. We evaluated several important mitochondrial dynamic markers, including mitofusin-1, mitofusin-2, dynamin related protein 1 (Drp-1) and optic atrophy 1 (OPA1). Surgery-induced an increase of mitofusin-2 levels (interaction: *F*_(1, 28)_ = 8.425, *p* = 0.0071; laparotomy factor: *F*_(1, 28)_ = 14.28, *p* = 0.0008; exercise factor: *F*_(1, 28)_ = 1.596, *p* = 0.2169. Multiple comparisons: 0.8668 ± 0.1835, *p* = 0.0004; Fig. 8b), and this up-regulation was reduced by resistance exercise (− 0.5405 ± 0.1835, *p* = 0.0379; Fig. 8b). No changes were demonstrated for the other markers. Mitofusin-2 is responsible for tethering the endoplasmic reticulum to mitochondria (ER–mitochondria contacts) that allow Ca^2+^ influx from the ER to mitochondria. From our TEM observation, there was a significant increase of ER-mitochondrial contact in the laparotomy group compared with control (interaction: *F*_(1, 76)_ = 9.632, *p* = 0.0027; laparotomy factor: *F*_(1, 76)_ = 12.4, *p* = 0.0007; exercise factor: *F*_(1, 76)_ = 14.4, *p* = 0.0003. Multiple comparisons: − 0.7114 ± 0.1519, *p* < 0.0001, Fig. 8c, d). Preoperative resistance training significantly reversed this surgery-induced contact (− 0.7407 ± 0.1519, *p* < 0.0001, Fig. 8c, d). These data showed that apart from mitochondrial deficits, preoperative resistance training also improved postoperative cognitive decline in aged mice by modulating the mitochondrial dynamic (contact with ER) in the aged mice.

Synapses are sites of high-energy demand that are particularly vulnerable to mitochondria dysfunction. Synaptosomes were isolated from the hippocampus and there was a significant increase of phospho-NR2A at tyrosine 1246 following surgery (interaction: *F*_(1, 17)_ = 8.946, *p* = 0.0082; *F*_(1, 17)_ = 12.49, *p* = 0.0026; *F*_(1, 17)_ = 1.451, *p* = 0.2449. Multiple comparisons: − 4.422 ± 0.9373, *p* = 0.0010; Fig. 8e and f) and phospho-NR2B at tyrosine 1472 (interaction: *F*_(1, 17)_ = 6.592, *p* = 0.0200; laparotomy factor: *F*_(1, 17)_ = 2.191, *p* = 0.1571; exercise factor: *F*_(1, 17)_ = 3.088, *p* = 0.0969. Multiple comparisons: 1.547 ± 0.5286, *p* = 0.0424, Fig. 8e and f). The level of phospho-AMPK-α at Thr172, which can be activated by excessive NMDA receptors activity, was enhanced following surgery (interaction: *F*_(1, 17)_ = 6.955, *p* = 0.0173; laparotomy factor: *F*_(1, 17)_ = 1.919, *p* = 0.1838, *F*_(1, 17)_ = 4.573, *p* = 0.0473. Multiple comparisons: 0.4136 ± 0.1422, *p* = 0.0439; Fig. 8e and f). These laparotomy-induced changes were prevented by resistance training (p-NR2A: − 0.2843 ± 0.9373, *p* = 0.0343; p-NR2B: − 1.653 ± 0.5286, *p* = 0.0284; p-AMPK-α: − 0.491 ± 0.1422, *p* = 0.0146; Fig. 8e–g). These observations revealed that resistance exercise may prevent surgery-induced NMDA receptor and AMPK activation, thus improving postoperative cognitive function in aged mice.

## Discussion

Research into exercise to improve brain disorders is not novel, but the use of “preconditioning” resistance training against PNDs is far and few between. This study showed that resistance exercise improves postoperative brain health from several standpoints, the most important of which is its effectiveness in aged animals. The elderly and those with mild cognitive impairment (MCI) may have baseline deficits and neuroinflammatory tendencies that can be potentially reduced by this mode of training. It can confer cardiovascular as well as putative neurocognitive benefits perioperatively.

We have shown that compared with middle-aged counterparts, aged mice have increased body weight, higher basal levels of inflammatory markers, lower muscle strength and inferior aversive memory and hippocampal-dependent spatial learning. These risk factors for PNDs were reduced by a period of resistance exercise. Given that obesity [38] and pre-existing cognitive impairment [39] are independent risk factors for PNDs, our data indicate that resistance exercise might reduce the incidence of postoperative cognitive decline. Accordantly, preoperative resistance exercise improved surgery-induced adverse effects including cognitive impairment, synaptic deficits and neuroinflammation. Further we report a novel finding regarding neuroinflammation and mitochondrial dysfunction that might form a vicious circle which ultimately may lead to synaptic dysfunction and cognitive dysfunction. This dysfunction can be relieved by resistance exercise, possibly by mediating the PGC1-a/BDNF pathway.

Pre-existing cognitive impairment is a major risk factor for PNDs that occur in 20% and 35% of elderly patients undergo total hip joint replacement [39] and CABG [40], respectively. Consistent with previous reports [41], we demonstrated pre-existing cognitive impairment in 18-month-aged mice compared to middle-aged mice, and this pre-existing cognitive impairment was attenuated by resistance exercise (Fig. 1h and i). Moreover, postoperative cognitive decline was also prevented by resistance exercise pre-conditioning in aged mice (Fig. 2e–f), while adult mice showed no postoperative cognitive change. We also noticed that the beneficial effect of resistance exercise on aged mice dissipates when training stops, since there is no longer a difference between the control and active groups at 14 days after stopping training. Dendritic arborization and spine density are morphological features closely associated with synaptic plasticity, which underlies dynamic changes in neuronal circuits and is fundamental to learning and memory [42]. Resistance training prevented the postoperative decrease in dendritic process complexity and spine density in the DG of the hippocampus (Fig. 3), thus conferring beneficial effect on perioperative cognitive function and synaptic plasticity.

Increasing data suggest “inflamm-aging” [43] and MCI [44] contribute to the increased risk of PNDs. More importantly, elevated inflammatory cytokine levels correlate with the increased risk of cognitive decline with aging [45] and progression from MCI to AD [46]. Our results suggest resistance exercise as a promising non-pharmacological approach to suppress both peripheral and neuroinflammation in aged mice, by reducing pro-inflammatory cytokines IL-1β and TNF-α in the liver and increasing the anti-inflammatory cytokine IL-10 in the hippocampus (Fig. 4a). Although we did not reveal any change of IL-1β, TNF-α and IL-10 on postoperative day 1 resistance exercise was shown to reduce the elevated mRNA level of MCP-1 in the hippocampus following laparotomy (Fig. 4b). This differs from several studies that showed significantly increased expression of IL-1β and TNF-α [47], but consistent with the data from our previous study [22]. It has been suggested that astrocytes actively modulate immune responses in the CNS and evoke microglial activation via MCP-1-CCR2 signaling in surgery-induced cognitive dysfunction and neuroinflammation [32]. Gliosis is frequently observed in different neurocognitive disorders including AD and vascular dementia [48]. These reactive astrocytes are shown to produce neurotoxic and neuroinflammatory factors which contributes to neuronal and synaptic dysfunction [48]. In this study, a prolonged postoperative neuroinflammatory response is shown by the presence of increased numbers and hypertrophy of glia (Fig. 5). With preoperative training, activation of astrocytes and microglia in the postoperative mice was attenuated. These data suggested that our resistance training protocol may ameliorate surgery-induced cognitive dysfunction partly through inhibiting gliosis.

An acute inflammatory response may resolve after triggering cell regeneration and wound healing, or develops into a chronic response that can lead to progressive cell and organ dysfunction. The anti-inflammatory effect of resistance exercise seen in this study can be attributed to the secretion of myokines from contracting muscles (Fig. 6a–c) and changes in body composition (Fig. 1d–e). The myokine FGF-21 promotes “browning” of white adipose tissue in mice in response to exercise [49], thereby improving body composition and suppressing inflammation in obese individuals. Another myokine IL-6 can reduce inflammation by inducing IL-10 and suppressing TNF-α production [50]. Adipose tissue secretes pro-inflammatory adipokines during physical inactivity and in metabolic diseases [51]. The loss of visceral adipose tissue following exercise is IL-6 dependent [52]. Hence, myokines can contribute to the anti-inflammatory effect of resistance exercise directly by modulating myokine secretion and/or indirectly by reducing the source of pro-inflammatory adipokines.

We demonstrated a significant increase of resistance exercise induced elevation of PGC-1α both in liver and hippocampus. PGC-1α is one of the better characterized myokines that can induce hippocampal BDNF by regulating neuronal Fndc5 gene expression (35). We also demonstrated an inhibition of BDNF/Akt/GSK-3β signaling and an increased Bax/Bcl-2 ratio 24 h after surgery, which were attenuated by resistance exercise (Fig. 6d–f), and this effect on PGC-1α/BDNF signaling was still present on postoperative day 14 (Fig. 6g–h). Although we did not produce data demonstrating a causative relationship between the up-regulation of myokines and the BDNF signaling in this study, previous reports have demonstrated that endurance exercise induces hippocampal BDNF expression through the secretion of the peripheral myokine PGC-1a/FNDC5 [35]. These observations are in line with our findings that after resistance training of significant up-regulation of PGC-1a in the periphery and BDNF signaling in brain. This indicates the possibility that resistance training exerts neuroprotective effects in laparotomy model through BDNF signaling, which may depend on the secretion of myokines such as PGC-1a in our model.

PGC-1α has also been identified as a master regulator of mitochondrial biogenesis and the PGC-1α/BDNF pathway-mediated mitochondrial biogenesis and dynamic play important roles in the formation and maintenance of hippocampal dendritic spines and synapses [36]. Mitochondria are organelles in cells that widely known for their central role in supplying cellular energy [53]. In addition, mitochondria are involved in signal transduction, intracellular calcium regulation, and cellular differentiation, as well as regulating the cell growth and maintaining cell cycle. Recent studies indicated that mitochondria are pivotal to immune responses since they serve as both a target and source of innate immune signaling. Neuroinflammation has been shown to interfere with mitochondrial dynamic, membrane permeabilization, and mitophagy, resulting in mitochondrial dysfunction. Moreover, extracellular release of mitochondrial components exacerbates neuroinflammation, leading to a vicious inflammatory cycle and neuronal dysfunction. Hence preoperative resistance exercise mitigate post-surgical neuroinflammation through PGC-1α/BDNF pathway-mediated mitochondrial biogenesis and dynamic.

Evidence of deficits in mitochondrial biogenesis following surgery is supported by a reduced absolute mitochondrial number. Resistance exercise reduced the laparotomy-induced mitochondrial loss and mitochondrial damage, as indicated by the increased HSP60 in the cytosolic fraction and mitochondrial number, as well as decreased leakage of cytochrome *c* into cytosolic fraction percentage of damaged mitochondria (Fig. 7). However, we did not find any changes in the expression of OXPHOS complexes I–V in mitochondria fractions in the hippocampus (Additional file 3). In line with these results, no significant changes in HSP60 in the mitochondrial fraction of hippocampal tissues were observed between the animal groups. HSP 60 is an important mitochondrial chaperone which is responsible for maintaining mitochondrial function including OXPHOS activities [54]. These data implicated that surgery and resistance training affect mitochondrial function in an OXPHOS-independent manner.

Mitochondrial biogenesis and mitophagy, as well as mitochondrial fission and fusion, contribute to the overall balance of mitochondria elimination and synthesis, which have been shown to be pivotal to the exercise-induced mitochondrial adaptations and neuronal benefits [55]. The ER–mitochondria contacts enable highly efficient transfer of Ca^2+^ from the ER to mitochondria, which is essential to maintain normal mitochondrial metabolism. However, excessive ER–mitochondria Ca^2+^ influx will activate the mitochondrial permeability transition pore and induce leakage of cytochrome *c*, thereby triggering cellular apoptosis [56]. Zheng et al. have shown that DNA damage promotes the formation of ER–mitochondria contacts [57], thus facilitating Ca^2+^ influx and DNA damage-induced apoptosis. Resistance exercise might be protective to neurons in this regard as it reduced the formation of ER–mitochondria contacts in this experimental model (Fig. 8d). This hypothesis is supported by the post-surgical increase in mitofusin-2, a protein responsible for tethering ER to mitochondria [58], which was decreased by resistance exercise (Fig. 8b). However, the role of mitofusin-2 in this process needs further exploration as not only is it involved in other important mitochondrial functions [59], but also because of its controversial role in mediating ER–mitochondria contacts in other pathophysiological conditions [60, 61].

Normal NMDA receptors activity in the hippocampus is essential to synaptic plasticity [62]. Early studies have reported that over-activation of NMDA receptors is closely associated with excitotoxicity and is implicated in many neurological diseases [63, 64]. Subsequent evidence supports the “localization hypothesis” that espouse the dual nature of NMDA receptors based on their location [65]. Specifically, it has been proposed that the stimulation of synaptic NMDA receptors, mainly by up-regulation of NR2B subunit, attribute to neuronal health and longevity [65]. Interestingly, we demonstrated a trend of losing synaptic NR2B following surgery, which was prevented by resistance exercise (Fig. 8f).

Proteomic analysis of tyrosine phosphorylation of synaptic glutamate receptors, including α-amino-3-hydroxy-5-methyl-4-isoxazolepropionic acid (AMPA) receptors and NMDA receptors, found persistently upregulated NR2A-Y1246 and NR2B-Y1472 after brain ischemia [66]. Upregulation of NR2B-Y1472 was reported to be involved in excitotoxicity and contributed to brain injury [67]. Accordingly, we identified a significant increase in NR2A-Y1246 and NR2B-Y1472 after laparotomy (Fig. 8f) and activation of AMPK-α signaling, as indicated by the up-regulation of phosphorylated AMPK-α at Thr172. Both changes were prevented by preoperative resistance exercise. The activation of AMPK under times of energy stress can be both neuroprotective [68] and proapoptotic [69]. In animal stroke models, acute treatment with metformin, an AMPK-α agonist, has been shown to increase infarct volume; but reduce infarct volume with chronic administration [70]. Exercise has been well known to induce activation of AMPK signaling [71]. Given my exercise protocol lasted 5 weeks, the regulation of AMPK by resistance exercise in this study promoted neural survival, rather than neuronal death. In line with these findings, our data also indicated the up-regulation of AMPK pathway following surgery, while preoperative resistance training inhibited this AMPK activation. Taken together, our findings implicate the detrimental role of AMPK activation in postoperative animals which can be amended by resistance training.

There are several limitations to this study, the main one of which is its largely descriptive as it was exploratory in nature. As resistance exercise seems to exert a wide range of benefits it was difficult to isolate a target for mechanistic study by way of pharmacological antagonists or knockout model. We are not sure if it deactivated a single upstream signal or conferred benefit from a multitude of factors released. Consequently, it is not possible to ascertain whether resistance exercise and improved post-surgical cognitive performance was epiphenomenal or mechanistically related. It was not possible to determine if the improved postoperative performance was consequential to a raised baseline that prevented it from sliding down to a critical point in the vicious cycle of neuroinflammation, or due to a protective effect that is independent of the baseline. Another significant limitation is the lack of evaluation of the effect of resistance exercise in different age groups. Post operative neuroinflammation with accompanying cognitive impairment does occur in mice as young as 3 months of age, the effects of which benefit from anti-inflammatory measures [72]. Therefore, it is not unconceivable that resistance exercise may also confer perioperative benefits in this age group. However, further studies will be required to confirm whether this is indeed the case.

## Conclusions

We provided experimental evidence for wide-ranging and far-reaching benefits from prehabilitative resistance exercise, especially in improving the reserve and resilience of mitochondria against detrimental effects of surgically induced neuroinflammation and synaptic deficits. Although the specific mechanisms remain to be elucidated, this approach can be evaluated as an interim non-pharmacological measure in clinical settings.

## Supplementary Information

**Additional file 1.** Video of an aged mice undergoing resistance training. In this video, the mouse was trained to spontaneously climbed the ladder with 40% of its body weight attached to its tail.

**Additional file 2.** Methods, primer and antibody list.

**Additional file 3.** Expression of complex I-V in mitochondrial and cytosolic fractions. Representative blots and quantitative analysis of OXPHOS proteins, the intensity of band was normalized to that of VDAC and β-actin, respectively. Data presented as mean ± SEM and analyzed by two-way ANOVA test, followed by Tukey multiple comparisons test, n = 8, *p < 0.05, **p < 0.01.

## Data Availability

The data that support the findings of this study are available from the corresponding author upon reasonable request.
