# Supplementary material for: Prehabilitative resistance exercise reduces neuroinflammation and improves mitochondrial health in aged mice with perioperative neurocognitive disorders
Source: J Neuroinflammation. 2022 Jun 15;19:150. doi: 10.1186/s12974-022-02483-1 (PMC9199135; doi:10.1186/s12974-022-02483-1)
Supplement: Supplementary file 2 — Additional file 2. Methods, primer and antibody list. [file 12974_2022_2483_MOESM2_ESM.docx]

**Methods**

**Isolation of total lysates or cellular compartment fractions**

Whole protein lysates were prepared by mechanical homogenization in RIPA buffer containing protease and phosphatase inhibitors (Roche, Germany) on ice. After keeping on ice for 15 minutes, the homogenized samples were centrifuged at 12,000 × g for 15 minutes at 4°C. The supernatant was carefully isolated and stored in -80°C freezer for further use.

Freshly harvest samples were used for synaptosome extraction according to the manufacturer’s protocol. Briefly, the tissue samples were homogenized using a dounce grinder with ice-cold Syn-PER reagent (Thermo Fisher, Waltham, Massachusetts, USA) supplemented with protease and phosphatase inhibitors. After keeping on ice for 15 minutes, the homogenized samples were centrifuged at 1200 × g for 10 minutes at 4°C. The supernatant was carefully isolated and centrifuged at 15,000 × g for 20 minutes at 4°C. The pellet in the bottom of the tube contained the synaptosome and the supernatant represented the cytosolic fraction. They were carefully isolated and stored in -80°C freezer for further use.

Tissues were homogenized using a pre-cooled glass homogenizer in Mitochondria Isolation Buffer (Biovision, CA, US) supplemented with protease and phosphatase inhibitors according to the manufacturer’s protocol. After keeping on ice for 15 minutes, the homogenized samples were centrifuged at 600 × g for 10 minutes at 4°C. The supernatant was carefully isolated and centrifuged at 7000 × g for 20 minutes at 4°C, the pellet in the bottom was the mitochondria and the supernatant was the cytosolic fraction. They were carefully isolated and stored in -80°C freezer for further use.

## Table 1 The primer sequences and PCR conditions of exercise-induced factors and inflammatory cytokines

| Gene | Primer Sequences | Annealing  temperature |
| --- | --- | --- |
| Interleukin-1-β  (IL-1β) | F: 5’-CCTCCTTGCCTCTGATGG-3’  R: 5’-AGTGCTGCCTAATGTCCC-3’ | 60℃ |
| Tumour necrosis factor  (TNF-α) | F: 5’-CCCCAGTCTGTATCCTTCT-3’  R: 5’-ACTGTCCCAGCATCTTGT-3’ | 59℃ |
| Interleukin-6  (IL-6) | F: 5’-GGCAATTCTGATTGTATG-3’  R: 5’-CTCTGGCTTTGTCTTTCT-3’ | 56℃ |
| Fibroblast growth factor- 21  (FGF-21) | F: 5’-AGATCAGGGAGGATGGAACA-3’  R: 5’-TCAAAGTGAGGCGATCCATA-3’ | 60℃ |
| Interleukin-10  (IL-10) | F: 5’-CCAAGCCTTATCGGAAATGA-3’  R: 5’-TTCTCACCCAGGGAATTCAA-3’ | 60℃ |
| Monocyte Chemoattractant Protein-1  (MCP-1) | F: 5’-TGCTGTCTCAGCCAGATGCAGTTA-3’  R: 5’-TACAGCTTCTTTGGGACACCTGCT-3’ | 60℃ |
| Glyceraldehyde-3-phosphate dehydrogenase  (GAPDH) | F: 5’-ATTCAACGGCACAGTCAA-3’  R: 5’-CTCGCTCCTGGAAGATGG-3’ | 56℃ |
| Proliferator-activated receptor γ coactivator 1  PGC1-a | F: 5’-AACGATGACCCTCCTCACAC-3’  R: 5’- TCTGGGGTCAGAGGAAGAGA-3’ | 60℃ |

**Table 2 List of Antibodies**

| (a) Western blots | | | | | |
| --- | --- | --- | --- | --- | --- |
| No. | Antibody | Brand | Cat. No. | Dilution | Species /Incubation |
| 1 | Akt (Ser473) | CST | 4060S | 1:1000 | Rabbit, 4°C overnight |
| 2 | Akt | CST | 9272 | 1:3000 | Rabbit, 4°C overnight |
| 3 | p-AMPK-α (Thr172) | CST | 2535S | 1:1000 | Rabbit, 4°C overnight |
| 4 | AMPK-α | CST | 2532 | 1:1000 | Rabbit, 4°C overnight |
| 5 | Bax | CST | 2772S | 1:1000 | Rabbit, 4°C overnight |
| 6 | Bcl-2 | CST | 2876 | 1:1000 | Rabbit, 4°C overnight |
| 7 | BDNF | Santa Cruz Biotech. | Sc-546 | 1:1000 | Rabbit, 4°C overnight |
| 8 | COX IV (3E11) | CST | 4850 | 1:2000 | Rabbit; 4°C overnight |
| 9 | Cytochrome c (136F3) | CST | 4280 | 1:1000 | Rabbit; 4°C overnight |
| 10 | HSP60 (D6F1) | CST | 12165 | 1:2000 | Rabbit; 4°C overnight |
| 11 | Pyruvate Dehydrogenase | CST | 3205 | 1:1000 | Rabbit; 4°C overnight |
| 12 | SDHA (D6J9M) | CST | 11998 | 1:1000 | Rabbit; 4°C overnight |
| 13 | VDAC (D73D12) | CST | 4661 | 1:2000 | Rabbit; 4°C overnight |
| 14 | OPA1 (D6U6N) | CST | 80471 | 1:1000 | Rabbit; 4°C overnight |
| 15 | DRP1 (Ser616) (D9A1) | CST | 4494 | 1:1000 | Rabbit; 4°C overnight |
| 16 | DRP1 (D8H5) | CST | 5391 | 1:2000 | Rabbit; 4°C overnight |
| 17 | Mitofusin-1 (D6E2S) | CST | 14739 | 1:1000 | Rabbit; 4°C overnight |
| 18 | Mitofusin-2 (D1E9) | CST | 11925 | 1:1000 | Rabbit; 4°C overnight |
| 19 | ß-actin | Sigma | A5441 | 1:30000 | Mouse; RT, 1h |
| 20 | GAPDH | Sigma | G8795 | 1:20000 | Mouse; RT, 1h |
| 21 | Goat Anti-Rabbit Immunoglobulins/HRP | DAKO | P0447 | 1:400 | RT, 1h |
| 22 | Goat Anti-Mouse Immunoglobulins/HRP | DAKO | P0448 | 1:400 | RT, 1h |

| (**b**) IF staining | | | | | |
| --- | --- | --- | --- | --- | --- |
| No. | Antibody | Brand | Cat. No. | Dilution | Species /Incubation |
| 1 | Iba-l | Wako | 019-19741 | 1:400 | Rabbit; 4°C overnight |
| 2 | GFAP | Sigma | G3893 | 1:400 | Mouse; 4°C overnight |
| 3 | DAPI | Sigma-Aldrich | D9542 | 3μm | RT, 15 min; avoid light |
| 4 | Alexa Fluor 488 goat anti-rabbit | Invitrogen | A21069 | 1:400 | RT, 1h; avoid light |
| 5 | Alexa Fluor 568 goat anti-mouse | Invitrogen | Al 1019 | 1:400 | RT, 1h; avoid light |
