# Supplementary figures and images for: Prehabilitative resistance exercise reduces neuroinflammation and improves mitochondrial health in aged mice with perioperative neurocognitive disorders
Source: J Neuroinflammation. 2022 Jun 15;19:150. doi: 10.1186/s12974-022-02483-1 (PMC9199135; doi:10.1186/s12974-022-02483-1)

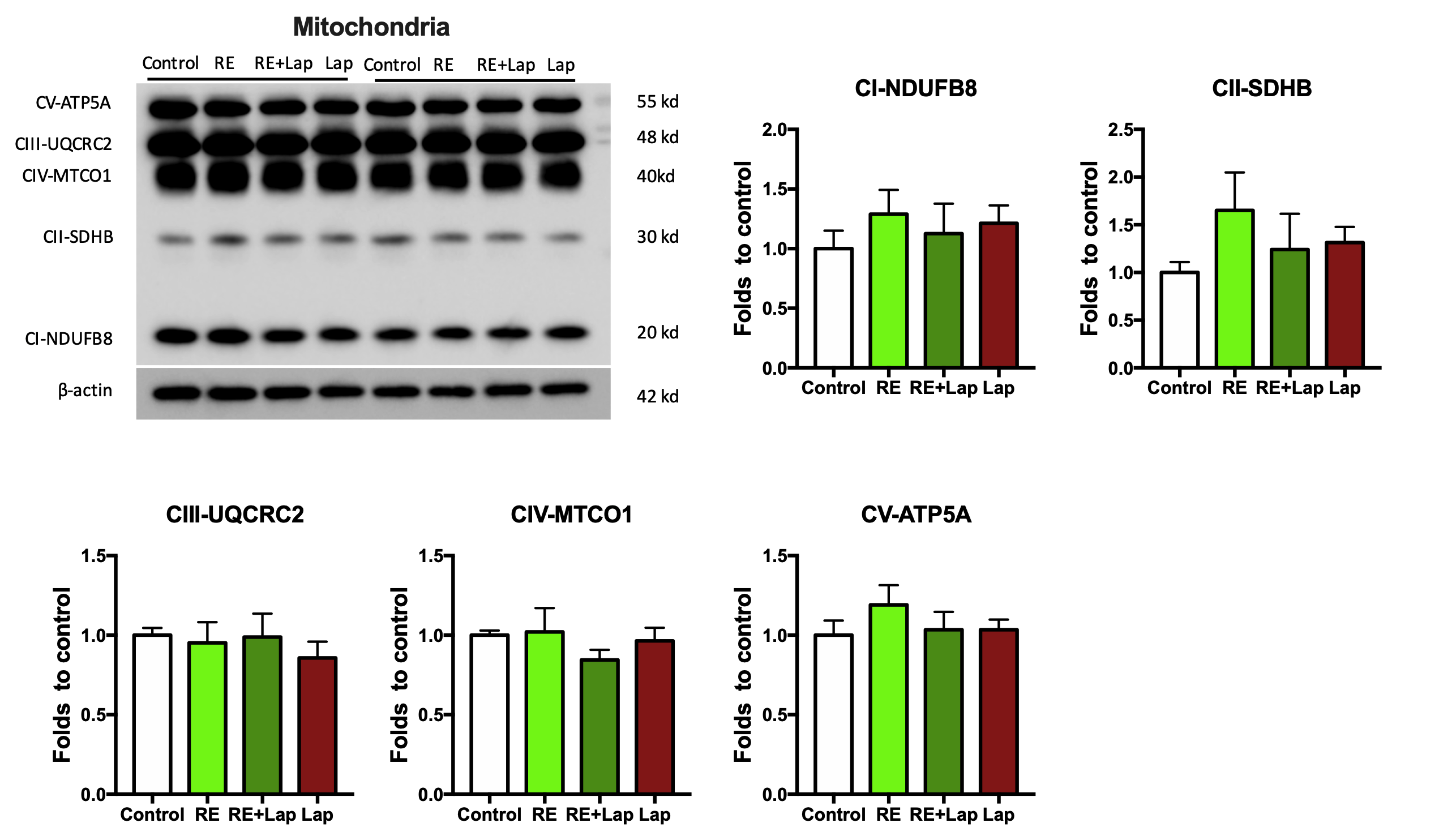

Supplement: Supplementary file 3 — Additional file 3. Expression of complex I-V in mitochondrial and cytosolic fractions. Representative blots and quantitative analysis of OXPHOS proteins, the intensity of band was normalized to that of VDAC and β-actin, respectively. Data presented as mean ± SEM and analyzed by two-way ANOVA test, followed by Tukey multiple comparisons test, n = 8, *p < 0.05, **p < 0.01. [file 12974_2022_2483_MOESM3_ESM.tiff]
